# The Interplay of Dietary Habits, Economic Factors, and Globalization: Assessing the Role of Institutional Quality

**DOI:** 10.3390/nu16183116

**Published:** 2024-09-15

**Authors:** Mohammad Naim Azimi, Mohammad Mafizur Rahman, Tek Maraseni

**Affiliations:** 1 School of Business, University of Southern Queensland, Toowoomba 4350, Australia; mafiz.rahman@usq.edu.au; 2 Centre for Sustainable Agricultural Systems (CSAS), University of Southern Queensland, Toowoomba 4350, Australia; tek.maraseni@usq.edu.au

**Keywords:** dietary habits, nutrition, institutional quality, economic growth, inflationary shock, I15, H51, O43

## Abstract

Background: Dietary habits are pivotal for population health and well-being, yet remain a pressing global issue, particularly in Sub-Saharan Africa (SSA), where economic instability and institutional challenges exacerbate dietary problems. Despite extensive research, there is a notable gap in the literature regarding the direct and interactive effects of institutional quality and inflationary shocks on dietary habits. Methods: This study delves into these complex interplays across 44 SSA nations from 2002 to 2022. Employing an innovative entropy method (EM) and the generalized autoregressive conditional heteroskedasticity (GARCH) modeling, the study introduces an inclusive institutional quality index and an inflationary shock predictor as crucial determinants of dietary habits in the literature. Results: The results from the panel-corrected standard error (PCSE) method and feasible generalized least squares (FGLS) model reveal that per capita GDP, school enrollment rate, government expenditures, globalization index, and urbanization are positively associated with population dietary habits, while inflationary shock, food insecurity, and unemployment rate exert negative influences. Notably, institutional quality acts as a catalyst, amplifying the positive effects of the former group and absorbing the negative impacts of the latter on population dietary habits. Additionally, a dynamic panel causality analysis confirms a bidirectional causality nexus between population dietary habits and all variables, except for inflationary shocks, which demonstrate a unidirectional causality link. Conclusions: These findings carry significant policy implications, underscoring the complex dynamics between institutional quality, inflationary shocks, and dietary habits in the region. The bidirectional causality highlights the need for holistic interventions that address economic, social, and institutional factors simultaneously. Moreover, the unidirectional causality of inflationary shocks on dietary habits suggests that stabilizing inflation is critical to protecting dietary habits. These results provide critical insights for policymakers to design targeted interventions aimed at improving nutrition, bolstering institutional frameworks, and ensuring public health resilience in the face of economic and social shocks.

## 1. Introduction

**Figure 1:**
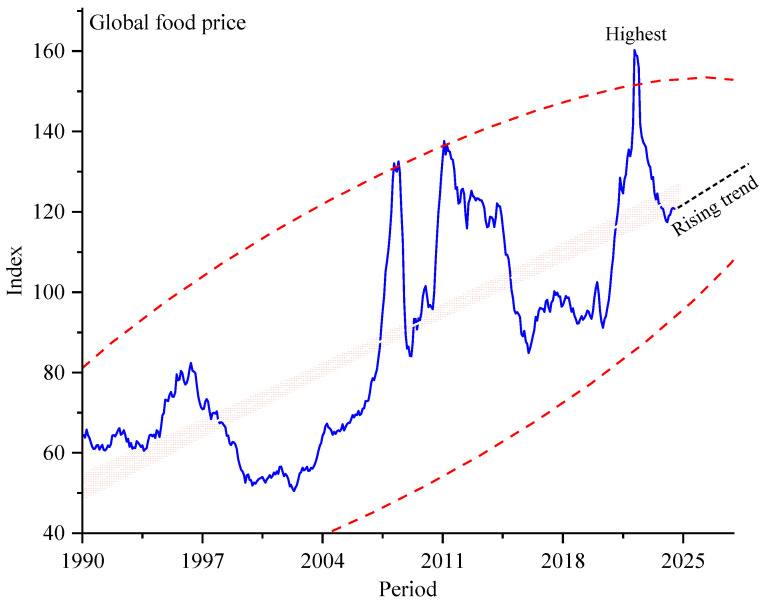
Historical global food price index from 1990 to 2024. Source: UN-FAO [5]; depicted by authors.

Accessing nutritious food is a fundamental human need to maintain a healthy and active life; however, it remains a pressing global concern, with over 43% of the world population lacking access to and affordability of a healthy daily diet [1]. As to recent statistics, Africa and Asia lead in the number of individuals unable to afford a sufficient and healthy diet, collectively contributing to 92% of the global increase in 2022 [2]. A global price shock, driven by the recent COVID-19 outbreak and escalating political tensions, has substantially affected the general costs of food items [3]. These disruptions resulted in a constrained supply chain and consistent overhangs in demand across the global population. Specifically, the outbreak has interrupted the normal course of production and supply chains, whereas existing geopolitical tensions and armed conflicts have intensified international trade barriers and commodity shortages, collectively contributing to heightened economic pressures on the growing percentage of individuals accessing essential food items [4]. As illustrated in Figure 1, the historical global food price index highlights notable volatility in food prices over time, with a spiking rise from 2019 to 2022 [5]. During this period, the index reached its highest level on record, reflecting a substantial upward shift with a rising trend thereafter. Literally, aside from economic constraints, there are various socio-economic factors that highly influence people’s affordability to maintain a healthy diet. These factors include escalating climate change, government investments in infrastructure, the agricultural productivity of a nation, the level of people’s food literacy, food insecurity, social status, and the degree of a country’s openness to globalization [6].

Presently, Sub-Saharan Africa (SSA), home to more than one billion diversified and rapidly growing populations, ranks first in poor dietary habits, resulting in severe health issues such as tangible stunted growth, significant malnutrition, faltered immune systems, and susceptibility to widespread diseases [7]. The Sustainable Development Goals (SDGs) agenda, particularly SDG-2, highlights the pressing need to address global malnutrition and hunger by the end of 2030. It aims to prevent the escalation of malnutrition and has far-reaching effects on health and social well-being. However, the reduction in severe malnutrition cases has not materialized as expected, and in fact, there has been a global increase of over 1%, with a greater proportion of this increase in the SSA region [8,9]. Although this catastrophe is primarily attributed to the region’s volatile economic performance, insufficient personal disposal income, growing climate change, and high rates of poverty and unemployment [10], weak institutional quality driven by heightened corruption and ineffective governance may be the hidden and yet underexplored aspect of this dire situation in the region [11].

Despite the considerable research on socioeconomic and demographic predictors of dietary habits in the SSA region, existing studies often overlook the influence of institutional quality and macroeconomic shocks such as inflationary pressures. Most studies, particularly those conducted by Weil et al. [12], Popkin [13], Zerbo et al. [14], Zeba et al. [15], Sodjinou et al. [16], Abizari and Ali [17], Cutler et al. [18], Galbete et al. [19], and Noort et al. [20], have concentrated on the impact of conventional factors like income, education, food security, and population growth on dietary habits. However, they have largely neglected the critical role of governance quality and the emerging influence of inflationary episodes in shaping these habits. Specifically, there has been little attention to how institutional setups moderate the relationship between dietary habits and key internal and external determinants. These gaps underscore the need to explore how existing governance frameworks can either exacerbate or alleviate the effects of economic and social shocks on dietary habits in the SSA. Addressing the specified gaps is essential, as institutional quality may play a crucial role in shaping dietary outcomes, particularly in SSA, where economic volatility and governance challenges are prevalent. To the extent of our knowledge, the available literature reports no empirical studies that have examined the direct and spillover effects of institutional quality and inflationary shocks on population dietary habits in the SSA. This underscores the need to examine how institutional quality and inflationary shocks impact dietary habits while accounting for major macroeconomic, globalization, demographic, and social indicators in the region. Therefore, the key objectives of the present examination are translated into four key research questions of the present time. Firstly, how does comprehensive institutional quality explain existing dietary habit predictors? Secondly, does institutional quality effectively modulate the relationships between dietary habits and key macroeconomic, social, globalization, and demographic factors? Thirdly, what is the magnitude and size of the impact of the inflationary shock on dietary habits in the region? Fourthly, is institutional quality significant in absorbing the negative impact of inflationary shock, food insecurity, and the unemployment rate on the subject?

The novelty of this piece of research lies in its comprehensive empirical methodologies to offering evidence-based answers to the formulated research questions, making significant contributions to the existing body of knowledge as follows: Firstly, considering the incremental patterns of food price inflation, this study employs an innovative approach by constructing an inflationary shock predictor using the generalized autoregressive conditional heteroskedasticity (GARCH) model, which captures volatility and inflationary shocks from regional data points. This methodology differs from previous approaches by accounting for the inherent variability and clustering of inflationary shocks, allowing for a more nuanced understanding of how these shocks influence dietary habits across the SSA region. Unlike the traditional static model, the GARCH-based inflationary shock predictor is uniquely suited to highlight the role of hyperinflationary episodes, thus providing policymakers with critical insights into the measures needed to stabilize economies and mitigate their adverse impact on the subject. For instance, by acknowledging the inflation rate as a stylized fact across all economies, the inflationary shock predictor offers deeper insights into the precise economic variations of the region in an open market environment—that is, open to both the pass-through and globally transmitted inflation shocks. This approach helps identify necessary measures for achieving macroeconomic stability to absorb pass-through and external shocks impacting existing dietary habits in the SSA. Secondly, as a counter predictor to potential negativities arising from various socioeconomic factors and inflationary shocks, the study innovatively develops a comprehensive institutional quality index using six measures of the Worldwide Governance Indicators and the intricate entropy method (EM), which allows for a more dynamics and holistic representation of existing governance quality compared to prior research that often relied on single indicator. Employing the institutional quality index as both an independent and modulating variable in the analysis helps highlight specific policy areas that need urgent interventions by policymakers in the SSA. For instance, it identifies how uplifting institutional setups can mitigate both internal and external negativities and amplifies the positive impact of improving factors on existing dietary habits. This strategy further paves the way for targeted interventions and effective policy reorientations. These two innovative approaches not only introduce novel methodological advancements to the contemporary literature but also enable a more precise evaluation of the research questions. By leveraging the inflationary shock predictor and the institutional quality index, this study offers a more holistic perspective, allowing for deeper insights into the complex interplay between economic, social, and institutional factors that influence dietary habits. Thirdly, given the critical state of dietary habits in the SSA and the lack of scholarly attention to this context in prior literature, the study prioritizes SSA nations to help policymakers in reorienting their existing strategies to address malnutrition issues from a distinct perspective. By using sophisticated analysis through the panel-corrected standard error (PCSE), feasible generalized least squares (FGLS), and the dynamic panel causality methodologies, this research addresses the issues of cross-sectional independence, heteroskedasticity, and potential autocorrelation among nations. These methods enable the exploration of nuanced relationships and causal links among the variables, offering actionable insights for targeted policy interventions.

The remainder of this article is organized as follows: Section 2 reviews the conceptual framework, current empirical literature, and highlights existing gaps in the literature. Section 3 presents the data and variables used in the study. It additionally explains the methods employed to construct new variables. Section 4 explains the methodology. Section 5 presents the empirical results. Section 6 briefly discusses the findings. Section 7 concludes the article, followed by specific policy implications, limitations of the study, and directions for future research.

## 2. Review of Literature

### 2.1. Conceptual Background

According to Medina et al. [21] and Sogari et al. [22], dietary habits are generally described as an act of conscious, repetitive habits that guide individuals in selecting and consuming specific foods and diets, influenced by social, cultural, and economic factors. These habits are deeply shaped by personal preferences and cultural norms, highlighting broader patterns within communities [23]. However, in the post-modern era, significant advancements in the food industry, coupled with a critical focus on subjective relativism, have led to increasing criticism of the classical concept of dietary habits. There is now a greater emphasis on the intricate causality between dietary habits and various economic, social, technological, and traditional stressors, acknowledging how these factors influence each other and fluctuate over time [24,25,26]. Additionally, traditional norms shape dietary patterns through diverse, natural ingredients that reflect the local availability and cultural heritage of a nation. Conversely, in modern norms, it is increasingly defined by the use of less natural ingredients that rely instead on high-tech machinery and artificial additives [27], leading to critical health issues. This shift from traditional to modern dietary practices represents a broader evolution in dietary habits globally [28], influenced by socioeconomic, technological, and cultural transformations. Traditional dietary practices characterized by the use of locally sourced ingredients and deeply embedded in cultural heritage, have been gradually altered by the rise of modern food production techniques. Modern dietary habits emphasize convenience, often relying on processed foods, artificial additives, and industrial-scale production. However, in response to increasing awareness of health and sustainability, a hybrid concept has emerged that blends elements of both traditional and modern dietary practices [29]. This mixed concept seeks to preserve the nutritional and cultural values of traditional diets while incorporating modern techniques to enhance efficiency and availability [30]. As such, this evolving concept of dietary habits underscores the dynamic nature of food consumption patterns, reflecting the ongoing interaction between rapid globalization, technological innovations, consumer protection, and specific societal challenges [31]. Nonetheless, this nuanced concept merit comprehensive examination, particularly in the context of developing nations where dietary habits are highly susceptible to the influence of these emerging factors.

### 2.2. Empirical Background

The burgeoning body of empirical literature on the novel factors affecting population dietary habits remains highly in flux, particularly with respect to inflationary shocks and institutional setups, with urbanization receiving relatively little attention. However, the existing literature is predominantly characterized by a conventional examination of social and economic indicators, which is comprehensively reviewed in this part and summarized in Table 1.

#### 2.2.1. Economic Factors-Dietary Habit Nexus

Among common factors, the literature reports that economic growth is evidently having significant impacts on dietary habits. For instance, Gerbens-Leenes et al. [32] examined the effects of per capita GDP on dietary consumption in a panel of 57 nations in European nations in 2001. Their results confirmed the positive correlation between dietary consumption and growth, highlighting differences in the effect size of growth varying according to a nation’s income level. Vanheuvelen and Vanheuvelen [33] analyzed the association between economic development, gender, and dietary habits to predict targeted health outcomes in 31 high- and middle-income countries using survey data for 2011 and a series of regression analyses. Their key results supported a heterogeneity between men’s and women’s dietary habits linked to their national economic development status. Bonaccio et al. [34] examined the effects of the economic crisis on Italians’ dietary habits from 2010 to 2013 using survey data from 9319 participants. Employing the logistic regression method, their study revealed a strong association between lower socioeconomic status, characterized by low income and unemployment, and poorer dietary habits compared to high-income, employed individuals. Furthermore, Raji [35] investigated the causality nexus between economic growth and nutrition intake in Nigeria from 1990 to 2013 using the vector error-correcting (VEC) approach and the impulse response function. The author noticed a long-term bidirectional causality among their employed predictors; however, only a unidirectional link has been found between growth and education. Moradi [36] utilized aggregate dataset from over 200,000 female participants across 28 SSA regions, with their heights as a predictor of nutritional status. The study observed a significant relationship between the level of economic development and women’s heights, evidenced by comparing the nutritional outcomes of women born in the 1960s cohort with those born in the 1980s, linked to economic development in the region. The review reveals that while these studies have explored the economic factors and habits nexus, a more detailed analysis with the most recent data and robust analysis in light of institutional quality is needed. Therefore, we develop the following hypotheses:

**Hypothesis 1:**  *Economic growth is instrumental in explaining the population’s dietary habits in the SSA region.*

**Hypothesis 2:**  *The effects of economic growth enhance with the modulation of institutional quality.*

#### 2.2.2. Unemployment–Dietary Habits Nexus

It is widely evident that unemployment serves as an economic force majeure, profoundly disrupting the population’s normal dietary habits. Milicic and DeCicca [37] delved into the impact of economic conditions proxied by the unemployment rate on dietary intake in Canada. Their results revealed that a heightened unemployment rate significantly reduces vegetable and fruit consumption across both education and gender status. Moreover, Smed et al. [38] assessed the influence of the unemployment rate on dietary composition in Denmark from 2008 to 2012 using a fixed effects model. Their findings highlighted both short- and long-run effects of the unemployment rate, leading to a significant shift in dietary habits from healthy, nutritious foods to increased consumption of sugar and carbohydrates. Likewise, Been et al. [39] examined the effects of the unemployment rate on average household food consumption patterns, measured by protein intake, in a panel of OECD nations from 1980 to 2020. The authors found that the unemployment rate significantly reduces the pattern of household food consumption across the bloc. Additionally, Bentolila and Ichino [40] investigated how households in the UK, Italy, the US, and Spain manage unemployment shocks in their food consumption. Their results demonstrated that food consumption patterns significantly change when family heads are unemployed. This effect is comparatively higher in the UK. Despite the limited number of studies addressing this pressing issue, the available literature underscores the need for more complex and comprehensive analyses to gain greater insights in the presence of institutional quality. To bridge this gap, we develop the following two key hypotheses:

**Hypothesis 3:**  *The unemployment rate is significantly detrimental to existing dietary habits in the SSA.*

**Hypothesis 4:**  *Institutional quality is effective in controlling the unemployment rate’s negativities on dietary habits.*

#### 2.2.3. Social Factors-Dietary Habits Nexus

Social factors play pivotal roles in shaping dietary habits within populations; however, there is no universally accepted quantitative standard for measuring their impact. Among numerous predictors, knowledge, and food literacy, along with food insecurity, are the most commonly used indicators to measure social factors, though empirical literature reports few studies examining their influence on dietary habits. Kohanmoo et al. [41] examined the effects of food insecurity on dietary habits among 190 women aged 20–55 years old in Iran. Using a cross-sectional analysis method, the authors observed that food insecurity is significantly associated with low-quality dietary habits among the panel. Similarly, Morales and Berkowitz [42] conducted a comprehensive review of numerous studies examining the relationship between food insecurity and dietary patterns. Their analysis consistently found that food insecurity has a universally negative impact on dietary habits, affecting both men and women alike. Mello et al. [43] investigated the impact of food insecurity on dietary habits across 1874 adults with low income. Their findings indicate that food insecurity results in a shift from nutritious foods to higher fat intake and increased juice consumption. Additionally, Nam and Suk [44] examined the link between food literacy and dietary behavior in Korea, analyzing data from 3321 participants. Employing basic statistical methods, they found that individuals with higher food literacy exhibited better dietary behavior compared to those with lower literacy levels. Amani and Soflaei [45] explored the effects of nutrition education on iron status and dietary habits in Iranian girls in high school. After two months, those who received education exhibited improved nutritional knowledge and mean corpuscular volume, whereas the control group showed a reverse behavior. Likewise, studies by Menor-Rodriguez et al. [46], Raut et al. [47], St. Pierre et al. [48], Li and Powdthavee [49], and Wei and Sun [50] have employed diverse proxies to measure the impact of literacy on dietary habits across various developed and developing economies. These studies collectively found a positive association between food literacy and improved dietary habits. Despite the abundance of studies in the literature, two gaps remain unaddressed: the role of institutional quality on the effects of food literacy on dietary habits, and the need for more complex examinations. To address these concerns, we formulate the following hypotheses:

**Hypothesis 5:**  *Food insecurity is detrimental to dietary habits; however, institutional quality is effective in absorbing its impacts.*

**Hypothesis 6:**  *Food literacy improves dietary habits, with higher impacts by the modulation of institutional quality.*

#### 2.2.4. Urbanization-Dietary Habits Nexus

Rapid urbanization has affected nearly every aspect of human life, particularly dietary habits. The available literature reports some studies addressing this critical issue. For instance, Colozza et al. [51] investigated the effects of urbanization on dietary patterns in Indonesia from 2000 to 2015 using logistic regression analysis. Their findings confirmed that urbanization highly impacts dietary habits, with a marked increase in the consumption of ultra-processed and soft drinks in urban cities. This shift in pattern contributes to less healthy dietary habits leading to severe health issues. Additionally, Ren et al. [52] examined the association between urbanization and dietary habits in rural areas of China from 2004 to 2011 using fixed effects and random effects methods. The authors observed that as urbanization increases, rural residents generally experience lower average calorie intake alongside improved dietary habits. Higher urbanization leads to a reduction in carbohydrate consumption and fat intake beyond a threshold level. Lazarou and Kalavana [53] employed a cross-sectional, semi-quantitative analysis studying the effects of urbanization on dietary habits among 1140 Cypriot kids. The authors observed insignificant differences between rural and urban kids; however, rural kids were found to have comparatively less junk food consumption and higher eating habits with their families. Likewise, Bellundagi et al. [54] evaluated the influence of urbanization on nutritional outcomes among 1275 girl children in India using multinominal logistic regression analysis. Their results highlighted that urbanization is positively associated with the dietary outcomes of women and girls. Although the available literature on the effects of urbanization on dietary habits, particularly in the SSA region, is relatively sparse, we address this issue from a distinct perspective by formulating the following hypotheses:

**Hypothesis 7:**  *Urbanization is a significant determinant in influencing dietary habits in the SSA.*

**Hypothesis 8:**  *Institutional quality instrumentally improves the relationships between urbanization and dietary habits.*

**Table 1:** Summary of the relevant literature.

Authors	Context	Determinant	Period	Method	Key Findings
Jones et al. [55]	India	Globalization	2019	CS	Globalization shifts adolescent’s food choices towards non-local, processed items.
Chang et al. [56]	China	Urbanization	1997–2011	CS	Urbanization improves dietary habits, but environmental impacts increase dietary costs.
Penne and Goedemé [57]	Europe	Income growth	2023	FA	Low income is a key barrier to accessing a healthy diet in Europe.
Bin Zahara et al. [58]	United States	Food security	2020	FC and P	Food security and attitude significantly influence dietary habits, moving towards more sweety and salty snack patterns.
French et al. [59]	Chicago	Household income	2019	CS	Higher-income households have better diet quality, purchasing more vegetables and dairy than lower-income households.
Vilar-Compte et al. [60]	SR	Poverty	68 papers	SR	Urban poverty and food insecurity are two critical factors affecting the quality of dietary habits.
Namirembe et al. [61]	Nepal	Nutrition governance	2021	GEE	Stronger nutrition governance leads to better child growth through healthy dietary habits.
Stone et al. [62]	United Kingdom	Inflation rate	2023	CS	The cost of living crisis results in poor dietary habits and higher food insecurity.
Heady and Ruel [63]	Panel data	Food CPI	2021–2022	CS	Food inflation increases the risk of child wasting and stunting in developing countries.

Notes: CS: cross-sectional, FA: factorial analysis, FC and P: frequency counts and percentage, SR: systematic review, generalized estimating equation, CPI: consumer price index.

### 2.3. Empirical Insights

Although recent empirical studies have made valuable contributions by highlighting the influence of conventional predictors of dietary habits, they often lack policy orientation and methodological rigor. Our in-depth review of the literature identifies three significant gaps: first, while common macro- and socio-economic indicators are frequently analyzed, the pass-through effects and externalities of growing inflationary shocks are often overlooked, leading to an incomplete understanding of dietary habit transition, particularly in the SSA region. Second, the role of institutional frameworks—both structural and process governance—in shaping public dietary habits is underemphasized, resulting in a more general rather than precise and in-depth understanding of dietary habits in SSA. Third, the influence of various economic sectors on dietary habits and the moderating role of institutional quality in this context are clearly neglected. By addressing these gaps, our study aims to advance the contemporary body of knowledge by providing new insights into these underexplored areas and offering novel, policy-oriented methodologies for more effective interventions.

## 3. Data and Variables

### 3.1. Data

The annual dataset utilized in this study spans the period from 2002 to 2022, primarily determined by the availability of data from reliable sources. Our preliminary screening revealed that institutional quality indicators, a critical variable in our analysis, are only available within this range. Since the study utilizes a large sample of countries, a 21-year panel dataset is sufficient to yield reliable results. Consequently, we have structured our panel dataset to be balanced across the specified period. This approach ensures that the analysis is based on consistent and credible data, allowing for more robust and reliable findings. The analysis focuses on Sub-Saharan African (SSA) countries, with a comprehensive list of these nations detailed in Table 2. The choice of SSA is mainly driven by four factors. Firstly, despite numerous studies exploring the relationship between dietary habits, economic growth, and health outcomes across diverse regions, SSA remains relatively underexplored in the literature [64]. Secondly, the prevailing assumption of a contemporaneous link between dietary habits, socioeconomic indicators, and emerging factors often leads to flawed policy implications when the critical role of institutional setups in countries with weak governance structures is overlooked [65]. Thirdly, SSA, characterized by its dynamic growth patterns, high rates of poverty, and unemployment offers a more realistic and nuanced view of the contemporary impact of key socioeconomic indicators and institutional predictors on dietary habits [66]. Fourthly, SSA’s high prevalence of malnutrition, limited dietary diversity, cultural dietary practices, urbanization impacts, agricultural productivity challenges, and globalization factors collectively provide a comprehensive context for examining the complex interplay between dietary habits, and socioeconomic, and institutional predictors.

### 3.2. Variables

#### 3.2.1. Dependent Variables

Our dependent variable, population dietary habits (PDH), is measured through three critical food supply metrics. The first variable, per capita kilocalorie supply from all foods (PK), captures the total daily food supply available to the population [67,68], reflecting both overall nutritional availability and, to a certain extent, the actual consumption pattern [9]. The second variable, per capita protein supply from all foods (PP), measures the daily availability of total protein to the population, in grams, encompassing both animal- and plant-based protein sources. The third variable, per capita fat supply from all foods (PF), also measured in grams, captures the availability of dietary fat to the population. Together, these three predictors provide a comprehensive overview of the population’s dietary habits, independent of health outcomes, allowing for an in-depth analysis of nutritional dynamics across the region.

#### 3.2.2. Independent Variables

Under the mixed concept, PDH is influenced by a multitude of factors, including economic, social, globalization, inflationary shocks, demographic, and institutional quality dimensions. Per capita GDP, measured in constant 2015 USD, is used to represent economic growth (EG). It captures the variations in personal disposal income and its impact on PDH. Although limited in numbers, recent studies by Burggraf et al. [69], Muhammad et al. [70], and Marques et al. [71] have examined the relationship between EG and PDH across diverse economies, highlighting the significant influence of income fluctuations on nutritional patterns and food consumption behavior. The unemployment rate (UR) expressed as an annual percentage represents the second macroeconomic indicator used to assess its impact on PDH. This metric is crucial because a higher UR is typically associated with an increased poverty rate and lower per capita income, which subsequently restricts access to nutritious food and alters typical consumption patterns [38,72].

**Figure 2:**
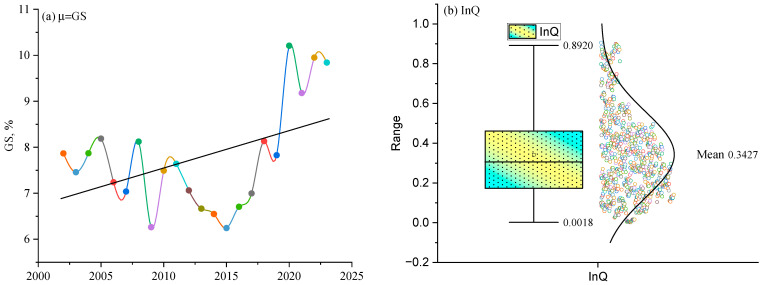
Constructed GS and InQ for SSA. Source: authors’ depiction.

School enrolment rate (SR), measured as the gross annual percentage of the total school enrolment serves as a key predictor of social factors that may influence PDH. A higher SR can enhance access to education, thereby improving nutritious knowledge and awareness. This improved understanding informs healthier food choices, positively impacting PDH. Studies conducted by Santos et al. [73], Nwani [74], and Raghupathi and Raghupathi [75] have also employed similar predictors to examine the impact of social factors, particularly general knowledge, on public health outcomes. Additionally, food insecurity (FI), a multifaceted socioeconomic indicator, is assessed by the prevalence of undernourishment. FI represents the percentage of individuals experiencing inadequate food intake in SSA to sustain a typical, active life [76,77]. As a pivotal determinant of PDH, FI profoundly impacts nutritional quality by restricting access to sufficient food. Moreover, recent political tensions and armed conflicts between certain nations have caused significant economic variations across the globe [78,79]. To account for the price shocks impacting PDH, we introduce an inflationary shock (GS) predictor derived from the inflation rate. The GS is modeled using the proposed generalized autoregressive conditional heteroskedasticity (GARCH) approach of Bollerslev [80], which is adept at capturing the volatility clustering and varying levels of economic instability specific to SSA. The GARCH model used is specified as follows:

(1) $$\begin{array}{l} \tau_{t}=\mu +\varepsilon_{t}\\ \varepsilon_{t}=z_{t}\sqrt{h_{t}}\\ h_{t}=\theta_{0}+\theta_{1}\varepsilon_{t-1}^{2}+\delta_{1}h_{t-1} \end{array}$$

where τ represents the inflation rate at the time t, μ is the mean value of τ, $\varepsilon_{t}$ indicates the error term, $z_{t}$ denotes the white noise process, $h_{t}$ is the conditional variance, and $\theta_{0}$, $\theta_{1}$, and $\delta_{1}$ are the parameters of the model (Appendix A details the estimation). Part (a) of Figure 2 depicts the annual average of the constructed GS for the SSA region. Given the importance of inflationary shocks as a predictor in our analysis, GARCH model is particularly well-suited for this purpose because it effectively models time-varying volatility and captures the persistence of inflationary shocks over time. The GARCH model, therefore, is ideal for capturing the true patterns of inflationary episodes, ensuring that our constructed predictor accurately reflects the dynamic nature of the data points.

Additionally, SSA suffers from weak institutional quality, impacting nearly all sectors [81,82]. This necessitates a comprehensive evaluation of how institutional quality affects PDH outcomes to inform effective policy and interventions. To this end, this study constructs a composite institutional quality index (InQ) following the advanced entropy method (EM) proposed by Shannon [83]. This approach, extensively utilized in the prior literature [84,85,86,87], offers several significant advantages. Firstly, unlike common methods, EM generates an index with ratio values, which facilitates a more nuanced and efficient analysis [88]. This ratio-based index is particularly useful for comparative studies and complex longitudinal analysis. Secondly, the methodology effectively circumvents the issues of negative values and benchmark overlays, providing a more robust and interpretable evaluation [89]. This ensures that the index is aligned with real-world institutional dynamics and offers a clearer representation of institutional quality in a country. Thirdly, this index quantifies institutional quality on a scale from 0 (worst-case scenario) to 1 (ideal point). This range not only simplifies interpretation but also allows for a consistent framework to monitor changes in institutional quality over time. Using Kaufmann and Kraay’s [90] Worldwide Governance Indicators (WGI), which include voice and accountability (VC), political stability (PS), government effectiveness (GE), regulatory quality (RQ), control of corruption (CC), and rule of law (RL), all expressed in percentile ranks ranging from 1 (low) to 100 (high), we construct the InQ through a simple three-step approach as outlined below:

Step-I: The WGI metrics are normalized to eliminate inconsistencies and overlying benchmarks across indicators. This can be completed through fitting the following equation:

(2) $$z_{WGI_{i}}=\frac{WGI_{i}-\min(WGI)}{\max(WGI)-\min(WGI)}$$

where $z_{WGI}$ represents the normalized values of WGI metrics, min and max are their minimum and maximum values.

Step-II: Based on the proportion of normalized values ($z_{WGI}$) from Equation (2), we apply the EM approach to assign appropriate weights to each WGI measures as follows:

(3) $$w_{i}=\frac{1-e_{i}}{\sum_{i=1}^{m}\left(1-e_{i}\right)}$$

where $w_{i}$ denotes the assigned weight to each indicator, derived from the proportion of the normalized values $\delta_{ij}=z_{ij}/\sum z_{ij}$ and the entropy $e_{i}=-k\sum \delta_{ij}ln(\delta_{ij})$ with $k=\frac{1}{ln(n)}$, where n represents the number of observations in our dataset.

Step-III: Aggregating the normalized indicators and the assigned weights, we can finally construct InQ as follows:

(4) $$\begin{array}{l} InQ_{j}=w_{VC}\cdot z_{vc_{j}}+w_{PS}\cdot z_{PS_{j}}+w_{RQ}\cdot z_{RQ_{j}}\\ \;\;\;\;\;\;\;+w_{GE}\cdot z_{GE_{j}}+w_{RL}\cdot z_{RL_{j}}+w_{CC}\cdot z_{CC_{j}} \end{array}$$

where $w_{VC}$, $w_{PS}$,…, $w_{CC}$ are assigned weights to WGI metrics and $z_{VC}$, $z_{PS}$,…, $z_{CC}$ are the normalized values of the indicators. Appendix B comprehensively outlines the estimation procedures, with part (b) of Figure 2 illustrating the constructed InQ.

In alignment with the emerging conceptual background, this study uses globalization to assess its effects on PDH. For this purpose, the globalization index (GI) proposed by KOF Swiss Economic Institute was developed, as detailed by Gygli et al. [91], is employed. The GI is expressed on a scale from 0 to 100, with higher values indicating greater openness of a country to globalization. Furthermore, urbanization (URB) is employed as a key variable to examine its impact on PDH. URB is measured as the percentage of the urban population relative to total population. Evidence shows that URB is a pivotal determinant of PDH, influencing dietary diversity, food availability, and lifestyle choice [92,93,94]. Finally, government expenditure (GX), measured as a percentage of GDP, is utilized to capture the dynamics of public spending impacting PDH. This metric is critical because a higher GX on health, education, social services, and infrastructure can highly impact PDH within a nation.

### 3.3. Sources of Compilation

The datasets relevant to per capita protein intake, per capita fat intake, and per capita kilocalories were sourced from the United Nations Food and Agriculture Organization [95]. Data for per capita GDP, unemployment rate, school enrollment rate, government expenditures, inflation rate, and prevalence of undernourishment were obtained from the World Development Indicators (WDI) provided by the World Bank [96]. The dataset for the globalization index was sourced from the KOF Swiss Economic Institute [91]. Additionally, data for variables used in constructing the institutional quality index were obtained from the Worldwide Governance Indicators [97].

## 4. Methods

### 4.1. Empirical Model

Our primary objective is to examine how the dynamics of economic growth, social factors, globalization, rising inflationary shocks, and institutional quality elucidate contemporary population dietary habits across the SSA region. Therefore, the following studies conducted by Dada and Ajide [98], Azimi and Rahman [99], and Abaidoo and Agyapong [100], the study commences with the specification of a long-run multivariate panel model as follows:

(5) $$\begin{array}{l} PDH_{it}=\delta +\theta_{1}EG_{it}+\theta_{2}UR_{it}+\theta_{3}SR_{it}+\theta_{4}GX_{it}+\theta_{5}GS_{it}\\ \;\;\;\;\;\;\;\;\;\;\;\;+\theta_{6}InQ_{it}+\theta_{7}FI_{it}+\theta_{8}URB_{it}+\theta_{9}GI_{it}+\xi_{i}+\varepsilon_{it} \end{array}$$

where *PDH* represents population dietary habits that include our key variables such as *PP*, *PF*, and *PK*, δ is the intercept, ξ denotes the unobserved country-specific effects, $\theta_{1}$ to $\theta_{9}$ are the long-run coefficients, and $\varepsilon_{it}$ is the error term, with all other variables described earlier. Equation (5) estimates the direct unconditional effects of the explanatory variables on *PDH*. However, to enhance our analysis, we further investigate how institutional quality (*InQ*) modulates the relationship between *PDH* and the explanatory variables. Specifically, we extend equation (5) by incorporating interaction terms between *InQ* and each explanatory variable (${Xs}_{it})$. This allows us to assess how variations in *InQ* affect the impact of the explanatory variables on *PDH*. The interaction terms are included by differentiating *InQ* with respect to each explanatory variable as follows:

(6) $$\frac{\partial PDH_{it}}{\partial Xs_{it}}=\theta_{6}+\varphi InQ_{it}$$

where φ represents the degree to which InQ enhances the effectiveness of economic, and social factors, environmental conditions, global inflationary shocks, and the control variables in influencing *PDH*. Having said so, we specify the following model:

(7) $$\begin{array}{l} PDH_{it}=\delta +\theta_{1}EG_{it}+\theta_{2}UR_{it}+\theta_{3}SR_{it}+\theta_{4}GX_{it}+\theta_{5}GS_{it}\\ \;\;\;\;\;\;\;\;\;\;\;\;\;+\theta_{6}InQ_{it}+\theta_{7}FI_{it}+\theta_{8}URB_{it}+\theta_{9}GI_{it}+\varphi (InQ_{it}\cdot Xs_{it})+\xi_{i}+\varepsilon_{it} \end{array}$$

Equation (7) estimates the moderating impact of *InQ* on the relationship between *PDH* and the explanatory variables, with other variables and coefficients remaining consistent as explained in Equations (5) and (6).

### 4.2. Estimation Procedure

**Figure 3:**
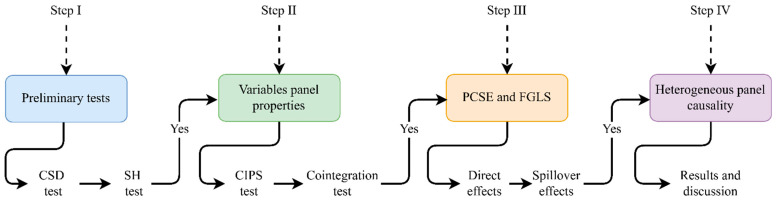
Research process. Notes: CSD: cross-sectional dependence, SH: slope heterogeneity, CIPS: cross-sectionally augmented Im, Pesaran, and Shin, PCSE: panel-corrected standard error, FGLS: feasible generalized least square.

The existing literature on the impact of socio-economic indicators on PDH reveals significant ambiguities in panel data analysis. Criticism often focuses on methodological shortcomings that neglect key empirical issues such as cross-sectional dependence (CSD) and slope heterogeneity (SH). In Section 2, we reviewed and noticed several dynamic panel estimation methods, including fixed effects (FE), random effects (RE), the generalized method of moment (GMM), and panel autoregressive distributed lags (ARDL) models that have been used by recent studies. Despite their widespread use, these models yield inconsistent results in the presence of CSD and SH [101,102]. FE and RE estimations tend to produce inconsistent and inefficient coefficients, especially when heteroskedasticity, autocorrelation, and CSD are present [103]. The GMM model is particularly well-suited for the analysis of panels with shorter time dimensions (T) and a larger number of cross-sectional units (N), provided that the data do not exhibit CSD and SH properties [104,105]. The cross-sectionally augmented autoregressive distributed lags (CS-ARDL) model developed by Chudik and Pesaran [106] is commonly used to address both CSD and SH issues. However, its reliability is contingent upon a sufficiently large sample size to allow for adequate lag selection, making it less consistent in studies with smaller samples, such as this one [107]. Additionally, Driscoll and Kraay [108] proposed a robust standard FE approach that accounts for both CSD and SH, although this model is generally more suitable for microeconomic estimations rather than broader macroeconomic policy analysis [109]. Therefore, in light of the aforementioned methodological challenges, this study adopts the panel-corrected standard error (PCSE) model as proposed by Beck and Katz [110]. The PCSE model is particularly adept at addressing serious econometric issues such as CSD, SH, serial correlation, and error heteroskedasticity in small panel samples. These issues often arise in empirical studies involving diverse economic indicators and addressing them ensures that the estimates are more reliable and robust. However, while the PCSE model is effective in generating consistent long-term estimates, it does have limitations in capturing short-run dynamics. Short-run estimates can be crucial for informing urgent policy interventions. Moreover, to enhance the robustness of the PCSE estimates, the study employs the feasible generalized least square (FGLS) method as a robustness check. FGLS is well-suited for correcting heteroskedasticity and serial correlation across panels, providing additional validity to our results. The choice of PCSE and FGLS approaches aligns with prior studies, such as Ikpesu et al. [111], Le and Nguyen [112], and Rahman et al. [113], which demonstrate their applicability to similar datasets. Moreover, the combination of PCSE and FGLS enables a more nuanced understanding of data, balancing long-term perspectives. Additionally, it is crucial to capture the causal relationship between PDH, economic, social, environmental, and institutional quality predictors to highlight more specific areas of policy concerns within SSA. To achieve this, the study adopts the Granger non-causality method of Dumitrescu and Hurlin [114], a model well-suited for addressing heterogeneous panel causality and providing reliable results. From this point forward, to ensure empirical replicability, we illustrate a comprehensive four-step estimation procedure in Figure 3. This framework provides a structured approach for testing the formulated research hypotheses, thereby ensuring the effective achievement of the study’s primary objectives.

As illustrated in Figure 3, the first step involves employing the CSD model proposed by Pesaran [115] and the slope heterogeneity test developed by Pesaran and Yamagata [116] to determine the presence of CSD and SH in the panel under review. Upon confirming the presence of CSD and SH, the second step involves determining the stationarity properties and long-run equilibrium among the variables. This is accompanied using the CIPS method of Pesaran [117] and Westerlund’s [118] panel cointegration tests, respectively. These advanced methodologies are specifically designed to detect true stationarity and long-run relationships in the presence of CSD and SH, ensuring a robust model specification. Using a sample comprising 44 countries with 21 observations each, and grounded in the theoretical assumptions, the study proceeds to the third step. Here, we estimate the direct unconditional and spillover effects of the explanatory variables on PDH (Equations (5) and (7)) using the PCSE and FGLS. In the fourth step, the study estimates the panel causality test developed by Dumitrescu and Hurlin [114] to identify any potential causal directional effects between PDH and the explanatory variables, ensuring a more comprehensive analysis.

## 5. Results

### 5.1. Basic Statistics

This study begins with some essential descriptive statistics reported in Table 2. The data reveals that the mean per capita protein from all foods (PP) is 41.443 g daily, with a maximum of 67.94 g across the region [119]. In contrast, the generally recommended daily protein intake ranges between 1.2 to 2.00 g per kilogram of body weight, underscoring significant undernutrition in SSA, with a standard deviation of 14.09 g indicating considerable variability in protein intake. This variability suggests disparities in dietary access and quality within the population, highlighting the need for targeted nutritional interventions. The mean per capita fat supply (PF) is 33.636 g per day, which falls short of the recommended 44–78 g daily. The standard deviation of 16.909 g highlights significant variability in fat intake, pointing to disparities in dietary access and potential nutritional inadequacies in the region. Additionally, the mean per capita kilocalorie supply from all foods (PK) is 2079 kilocalories per day, indicating regional undernutrition as it deviates from the recommended average of 2200–3000 kilocalories per day [120,121]. Moreover, the standard deviation of PK, which is 1522.11 kilocalories per day, highlights the significant disparity in food supply across the countries in SSA. Additionally, per capita GDP (EG) averages USD 1881.997, with a standard deviation of USD 255.1 across the bloc, reflecting a significant economic variability. The unemployment rate (UR) reaches a maximum of 37.85%, with a mean value of 8.18% and a standard deviation of 6.90%, indicating highly significant disparity across SSA, where some nations experience lower UR, while others face much higher levels. The school enrollment rate (SR) averages 99.722%, signaling progress that may help mitigate unemployment challenges. Importantly, the standard deviation of SR is 11.374%, reflecting consistent progress across SSA in improving SR throughout the period. Government expenditure (GX) is relatively modest, averaging 3.757% of the region’s GDP. Theoretically, lower GX suggests substantial economic welfare challenges, potentially hindering the ability of nations to address critical social and infrastructural needs. Moreover, the inflationary shock averages 1.623%, with an alarming peak of 597.28% and a standard deviation of 27.368%. This underscores severe economic vulnerability and highlights the potential adverse effects of inflationary pressures on nutritional stability and overall economic resilience in the region, as some nations in SSA face extreme inflationary pressures. The mean value of the globalization index is 46.649, with a maximum of 49.728. Despite this maximum, all nations in SSA still remain highly below the global average of 63.65.

As shown in Table 2, InQ in SSA is at a catastrophic level, with an average score of 0.343 and a minimum of 0.0018. An average score below 0.50 across all six measures of the WGI signifies inadequate governance performance, highlighting a pressing need for substantial improvement [122]. While the remaining statistics offer additional insights, the study advances to examine potential multicollinearity among the variables using both conventional correlation and variance inflation factor (VIF) approaches. Table 3 reveals that the correlation between the augmented variables is weak, as corroborated by the VIF results, which show an overall mean of 1.80 and individual VIF of below 5 (see, for instance, [123]). These findings imply that multicollinearity does not exist, allowing the study to proceed with incorporating all these variables into the subsequent regression analysis.

### 5.2. CD, SH, Panel Unit Root, and Cointegration

Table 4 delineates the outcomes of the CD and SH tests. The analysis elucidates that, apart from InQ, all other variables manifest reject the null hypothesis of no CD. This underscores that pervasive economic interconnectedness, shared trade dynamics, and similar consumption patterns contribute to the observed CD within SSA. These results align with the findings of Sassi [124], Katusiime [125], and Beyene [7], who have reported similar results for the SSA region. Moreover, the results obtained from the Pesaran and Yamagata [116] test reject the null hypothesis of slope homogeneity across Models 1–3. In this study, Model 1 pertains to the scenario where the dependent variable is PP, Model 2 applies when the dependent variable is PF, and Model 3 is used when the dependent variable is PK. The results imply that the slopes are not uniform and vary across the included countries, highlighting the need for meticulous model specification.

Additionally, Table 5 reports the results of the CIPS panel unit root test, as proposed by Pesaran [117], which is initialized by the cross-sectionally augmented Dickey–Fuller (CADF) estimates. The application of the CIPS and CADF is based on the confirmation of CSD and SH in Table 4, as conventional panel unit root tests are inadequate for accurately detecting the true stationarity of the variables in the presence of CSD and SH. Both CADF and CIPS methods are employed using constant and trend predictors. The findings unanimously indicate that GX, GS, and InQ are level stationary variables at a 1% significance level. In contrast, the remaining variables achieve stationarity only after first differencing. Although the variables follow a mixed integration order of I (0) and I (1), none of them displays a higher degree of integration. This underscores the need to further explore their long-run relationships using an appropriate cointegration method.

The long-run relationship among the variables in Models 1–3 is examined using both Kao’s [126] conventional cointegration approach and the advanced second-generation method proposed by Westerlund [118]. The results presented in Table 6 reveal that all three models exhibit significant cointegration with the incorporated explanatory variables, according to both methods. The significance of cointegration in the context of this study confirms the existence of stable and long-term relationships among the variables, meaning that despite short-term fluctuations, variables like EG, UR, SR, GX, GS, InQ, FI, URB, GI, and PDH move together in the long run. This implies that the effects of the explanatory variables on PDH are not merely temporary but persist over time, allowing us to infer the impacts of these explanatory variables on PDH as enduring and stable. These findings also warrant further exploration into the scale and magnitude of these long-term relationships.

### 5.3. PCSE and FGLS Estimates

Table 7 displays the direct unconditional impacts of the explanatory variables on PDH for Models 1–3. The results are obtained using the PCSE method, with additional robustness checks performed using the FGLS model. The results of both the PCSE and FGLS methods are consistent with a minor difference only in their t-statistics. While the results align with the anticipated coefficient signs, they reveal that EG, SR, GX, GI, and URB exert significantly positive impacts on PDH. Conversely, UR and FI are associated with negative impacts on PDH. Notably, InQ demonstrates a relatively more substantial positive influence on PDH, whereas GS exerts a detrimental effect. Specifically, the results highlight that a one-dollar increase in per capita GDP results in increases of 0.1884 g in per capita protein intake from all foods (PP), 0.2985 g of per capita fat from all foods (PF), and 0.443 per capita kilocalorie intake (PK). According to Engle’s Law—an economic theory—as per capita income increases, the proportion of income spent on food decreases, but the absolute quantity and quality of food consumed tends to improve. As a result, a higher income allows individuals to purchase more nutrient-dense foods, leading to increased intakes of protein, fat, and calories. Moreover, a 1% increase in UR leads to decreases of 0.449 in PP, 0.274 in PF, and 0.137 in PK, underscoring the detrimental impact of UR on nutritional intake in SSA. This implies that higher unemployment in SSA limits household income, reducing access to nutrient-rich foods and worsening overall nutritional intake. In contrast, the findings demonstrate that a 1% increase in SR leads to significant improvements in PP, PF, and PK by 0.122, 0.053, and 1.485 units, respectively. This outcome highlights the critical role of education and nutrition literacy in enhancing contemporary dietary habits within the SSA region.

Furthermore, the results reveal that a 1% increase in GX leads to increases of 1.948 g in PP, 0.131 g in PF, and 1.559 in PK, indicating that higher government spending positively influences dietary habits. Economic welfare theory emphasizes the crucial role of GX in enhancing a nation’s social and economic well-being. Higher GX supports improvements in nutritional quality and access, contributing to a healthier diet and overall better population health. Conversely, GS has detrimental effects on PDH, with a 1% increase in GS resulting in substantial decreases of 0.524 g in PP, 0.513 g in PF, and 0.568 kilocalories in PK. This indicates that increased inflationary shocks reduce the affordability and availability of nutrient-dense foods. As a result, SSA nations become more prone to consistent risks of undernutrition and compromised dietary quality. InQ, on the other hand, shows a significant positive impact, where a 1% improvement in the existing InQ results in increases of 4.521 g in PP, 2.064 g in PF, and 4.661 kilocalories in PK, highlighting its critical role in enhancing dietary habits. This underscores the pivotal role of robust nutrition governance and institutional quality in significantly enhancing dietary habits and overall nutritional intake in SSA. Food insecurity (FI) remains a critical challenge in the SSA region. The findings demonstrate a negative association between FI and PDH, showing that a 1% increase in FI results in decreases of 0.329 g in PP, 0.120 g in PF, and 1.593 kilocalories in PK. This finding implies that escalating FI directly diminishes access to essential nutrients, worsening malnutrition and contributing to more severe dietary deficiencies in SSA, where many already struggle with limited food resources.

Moreover, a 1% increase in URB leads to increases of 0.059 g in PP, 0.326 g in PF, and 0.178 kilocalories in PK. This suggests that urbanization enhances access to improved nutrition, as urban environments typically offer better employment opportunities, superior healthcare, and more diverse food options, thereby positively impacting dietary habits. Finally, GI has a positive impact on PDH. Specifically, a unit increase in GI results in increases of 0.606 g in PP, 0.720 g in PF, and 0.678 kilocalories in PK across the SSA region. These findings highlight that globalization drives greater availability and variety of foods, which can enhance nutrition and contribute to better dietary habits in the region.

In a bid to provide more critical insights and highlight specific areas of policy concerns, we estimate the moderating impact of InQ on the relationships between PDH and the incorporated explanatory variables. The results of the PCSE are presented in Table 8, Table 9 and Table 10. For effective space utilization, the robustness checks obtained from the FGLS model are reported in Table A1, Table A2 and Table A3 of Appendix C. The estimated coefficients of both the PCSE and FGLS are strongly identical, with only minor variations in their t-statistics, pinpointing appropriate specifications. The findings in Table 8 reveal that while EG alone increases PP by 0.1878 g, the presence of high institutional quality significantly enhances this effect. When moderated by InQ, the increase in PP jumps to 1.047 g, highlighting that robust institutions substantially amplify the nutritional benefits of economic growth. This underscores the critical role of strong institutional frameworks in not only supporting economic growth but also in maximizing its positive impact on dietary intake in SSA. Furthermore, the results show that the direct impact of UR on PP remains negative. A 1% increase in UR results in a decrease of 0.406 g in PP. However, when this relationship is moderated by InQ, this negative influence is significantly mitigated, reducing to just 0.087 g. This demonstrates the effectiveness of strong institutions in alleviating the adverse consequences of UR on dietary habits in a macro context. Likewise, the findings indicate that the moderating effects of InQ significantly improve the relationship between SR and PP. The direct impact of SR on PP results in a 0.160 g increase, but with the moderating impact of InQ, the effect size of SR on PP increases by an additional 1.592 g. This highlights that improved institutional quality enhances the benefits of increased SR on nutrition, pinpointing the importance of strong governance in translating educational gains into improved dietary outcomes in SSA.

Additionally, the moderating effect of InQ significantly enhances the efficacy of GX and URB on contemporary PP across the region. The direct impacts of GX and URB resulted in increases of 0.903 and 0.046 g in PP, respectively. However, when moderated by InQ, these effects are substantially amplified, leading to increases of 2.396 and 0.832 g in PP, respectively. Literally, robust institutions facilitate the effective channelization of government expenditures, reduce corruption, and improve governmental efficacy. This leads to improved spending on nutrition-related programs and better urban planning and implementation, ultimately resulting in improved dietary habits within the nation. Despite its detrimental impact on PP, as evidenced in Table 7, the negative effects of GS are largely mitigated by the moderating influence of InQ. While GS continues to negatively impact PP, the negative effect size is reduced to a mere 0.00058 g in PP when moderated by InQ. This signifies the role of robust institutional frameworks in buffering the population against the negative impact of external inflationary shocks on dietary habits. Similarly, the moderating effects of InQ are shown to be influential in absorbing the negative impact of FI on PP. While FI remains significant, InQ reduces its adverse impacts by 0.0007 g on PP. Furthermore, the findings reveal that the moderating impact of InQ on GI-PP is significant, indicating that as InQ enhances, the positive impact of GI on PP increases by 1.0007 g. This implies that robust institutional frameworks leverage higher openness to globalization in boosting nutritional outcomes in SSA.

Table 9 reports the moderating effects of InQ on PF and the explanatory variables. The results reveal that InQ significantly improves the relationship between PF and EG, resulting in an increase of 0.9223 g. Similarly, InQ is highly effective in enhancing the positive effects of SR, GX, and URB on PF, with increases of 2.006 g, 0.184 g, and 0.787 g, respectively. The results demonstrate that the direct effect of UR on PF still remains negative. Nevertheless, InQ shows substantial efficacy in mitigating this adverse impact, reducing it by 0.170 g. Likewise, while GS continues to exert a detrimental effect on PF, InQ significantly attenuates this impact, reducing the negative effect size to a minimal 0.0009 g. Additionally, InQ is instrumental in moderating the negative impact of FI on PF. Although FI maintains its significance, the moderating role of InQ significantly lessens its adverse impacts, reducing it by 0.155 g. Conversely, the moderating impact of InQ improves the relationship between PF and GI by 1.006 g.

**Table 10:** Moderating effects of InQ on PK-Xs nexus.

Variables	PCSE Model Estimates							
	InQ on PK–EG Nexus	InQ on PK–UR Nexus	InQ on PK–SR Nexus	InQ on PK–GX Nexus	InQ on PK–GS Nexus	InQ on PK–FI Nexus	InQ on PK–URB Nexus	InQ on PK–CO_2_e Nexus
EG	0.454 ***	0.448 ***	0.445 ***	0.452 ***	0.445 ***	0.436 ***	0.438 ***	0.447 ***
	(20.49)	(6.43)	(5.56)	(7.05)	(5.50)	(5.74)	(4.76)	(4.46)
UR	−0.147 ***	−0.147 ***	−0.158 ***	−0.177 ***	−0.123 ***	−0.174 ***	−0.1637 ***	−0.146 ***
	(−5.93)	(−5.14)	(−6.62)	(−7.80)	(−6.38)	(−4.23)	(−7.44)	(−3.15)
SR	1.409 ***	1.649 ***	1.772 ***	1.750 ***	1.7311 ***	1.7164 ***	1.8326 ***	1.719 **
	(3.07)	(4.69)	(3.00)	(2.76)	(4.01)	(7.61)	(4.04)	(2.28)
GX	1.082 ***	1.451 ***	1.287 ***	1.311 ***	1.470 ***	1.010 ***	1.350 ***	1.343 ***
	(13.09)	(12.45)	(12.02)	(11.20)	(12.31)	(16.80)	(12.32)	(13.69)
GS	−0.596 **	−0.626 ***	−0.572 ***	−0.549 **	−0.684 ***	−0.498 ***	−0.633 ***	−0.510 ***
	(−2.47)	(−2.95)	(−2.78)	(−2.53)	(−3.25)	(−2.85)	(−2.71)	(−2.58)
InQ	4.089 ***	4.034 ***	4.198 ***	4.077 ***	4.061 ***	4.017 ***	4.110 ***	4.094 ***
	(8.36)	(10.79)	(4.31)	(7.54)	(5.51)	(9.92)	(7.29)	(4.40)
FI	−1.589 ***	−1.582 ***	−1.560 ***	−1.752 ***	−1.620 ***	−1.206 **	−1.546 ***	−1.628 ***
	(−5.81)	(−6.55)	(−5.25)	(−7.97)	(−9.62)	(−4.30)	(−8.59)	(−5.05)
URB	0.181 ***	0.182 ***	0.208 ***	0.206 ***	0.177 ***	0.185 ***	0.181 *	0.149 ***
	(6.48)	(9.78)	(6.18)	(6.11)	(9.70)	(9.29)	(1.67)	(7.15)
GI	0.667 **	0.594 *	0.677 **	0.786 **	0.887 ***	0.879 ***	0.804 **	0.856 ***
	(2.41)	(1.73)	(1.31)	(2.32)	(3.14)	(9.53)	(2.53)	(8.17)
InQ × EG	0.949 ***							
	(5.13)							
InQ × UR		−0.0025 ***						
		(−9.10)						
InQ × SR			2.422 ***					
			(4.80)					
InQ × GX				2.857 ***				
				(6.97)				
InQ × GS					−0.0332 ***			
					(−4.51)			
InQ × FI						−0.00871 ***		
						(−6.83)		
InQ × URB							1.080 ***	
							(8.37)	
InQ × GI								1.0605 ***
								(4.95)
Constant	8.147 ***	2.359 ***	6.654 ***	8.549 ***	8.636 ***	9.900 ***	6.482 ***	5.458 ***
	(79.43)	(42.21)	(24.31)	(28.80)	(51.10)	(33.18)	(46.77)	(65.80)
***Post-estimations***								
Observations	924	924	924	924	924	924	924	924
R-squared	0.489	0.321	0.301	0.322	0.304	0.548	0.306	0.476
Number of units	44	44	44	44	44	44	44	44

Notes: z-statistics are presented in parenthesis. ***, **, and * indicate significance at 1%, 5%, and 10% levels, respectively.

Finally, Table 10 outlines the moderating impact of InQ on PK and the incorporated explanatory variables. The findings demonstrate that InQ significantly enhances the PK–EG nexus, leading to an increase of 0.495 g. InQ also markedly strengthens the positive impacts of SR, GX, and URB on PK, resulting in increases of 1.013 g, 1.775 g, and 0.899 g, respectively. Conversely, while the direct impact of UR on PK is notably negative, InQ effectively mitigates this adverse impact, reducing it by 0.1445 g. Likewise, although GS continues to negatively affect PK, InQ substantially attenuates this impact, lowering the negative impact size to just 0.0332 g. Moreover, InQ plays a crucial role in moderating the negative effects of FI on PK. Despite its continued significance, InQ significantly reduces its detrimental impact, with a decrease of 1.538 g. Finally, the moderating effects of InQ are effective in improving the relationships between GI and PK by 1.0605 g.

### 5.4. Panel Causality Analysis

The results detailed in Table 7, and Table 8, Table 9 and Table 10 provide compelling insights into the effects of the explanatory predictors on PDH (PP, PF, PK), particularly InQ. To further elucidate these connections, Table 11 presents the estimated results from the Dumitrescu and Hurlin [114] causality model across Models 1–3. The results reveal a compelling dynamic in the relationship between GS and PDH: a unidirectional causality flows from GS to PDH, suggesting that fluctuations in inflationary shocks significantly cause dietary outcomes, but dietary habits do not feed into inflationary changes. This underscores the critical influence of GS on PDH. In contrast, the bidirectional causality observed between other variables, such as EG with PDH, highlights the complex interplay where improvements in EG enhance dietary habits, which, in turn, can influence further socioeconomic development. This bidirectional relationship supports theories of reverse causation, where positive feedback loops between economic progress and nutrition create reinforcing cycles of improvement. Additionally, the bidirectional causality between GX and PDH suggests that increased government spending on nutrition improves dietary practices, while better nutrition encourages more investment in nutrition. This mutual reinforcement highlights the importance of sustained public spending to support and enhance nutritional outcomes.

Moreover, the bidirectional causality between FI and PDH shows that FI worsens dietary habit quality, and poor dietary habits can further exacerbate FI. This underscores the need for integrated strategies addressing both FI and PDH to break the cycle. The bidirectional causality between UR and PDH indicates that high unemployment can deteriorate dietary habits due to lower income, while poor nutrition can impair productivity and increase unemployment. Likewise, the bidirectional causality between InQ and PDH highlights that stronger institutions lead to better dietary habits through effective policies, and improved nutrition strengthens institutional capacity. Investing in institutional quality can enhance nutritional outcomes and vice versa.

## 6. Discussion

**Figure 4:**
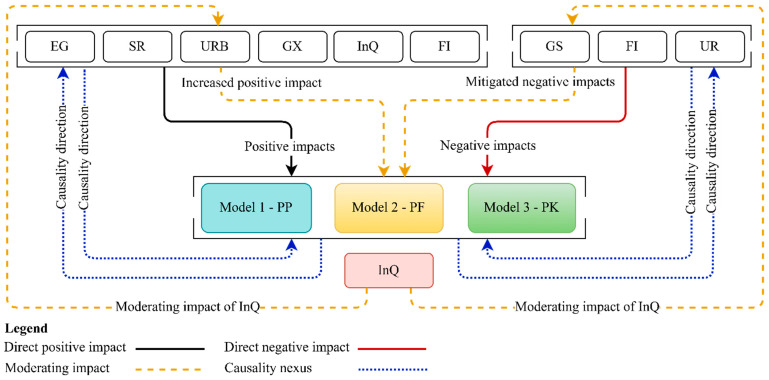
Summary of the results. Source: authors’ depiction.

Altogether, in the ongoing discourse surrounding the multifaceted impact of economic conditions, food insecurity, social factors, globalization, and demographic variables on malnutrition and their consequences on population dietary habits, certain pivotal aspects remain underexplored. Specifically, the impact of inflationary shocks on general nutritional costs and the role of institutional settings in shaping contemporary PDH have not been sufficiently examined in recent research. This study delves into those overlooked aspects within the context of 44 SSA countries from 2002 to 2022, guided by three critical research questions pertinent to contemporary nutrition issues. To maintain brevity, Figure 4 summarizes the key results. However, the preliminary analysis (Table 2) underscores severe nutritional deficiencies compared to the recommended levels, compounded by substantial volatility in GS and markedly poor InQ throughout the SSA region. Our initial results align with those reported by Hassan [127], Akinlo [128], Beyene [129], John-Joy Owolade [130], and Beyene [7], who also observed the volatility of nutritional deficiencies, the pervasive issues of weak institutional quality, and severe food insecurity in SSA.

Leveraging the panel properties of the augmented variables presented in Table 3, Table 4, Table 5 and Table 6, this study employed the PCSE and FGLS models to investigate the impact of the explanatory variables on three critical PDH predictors: PP, PF, and PK, as illustrated in Table 2. Our results (Table 7) support the beneficial effects of EG on PDH. This relationship is primarily linked to the fact that robust EG often leads to improved personal disposal income, enhanced access to diverse nutritious foods, and overall improved living standards, which collectively contribute to healthier dietary habits [71]. Prior empirical studies, such as those by Burggraf et al. [69], Lo et al. [131], Muhammad et al. [70], Mukhopadhyay and Thomassin [132], and Gerbens-Leenes et al. [32], have corroborated these findings. Upon further examination (Table 7, Table 8, Table 9 and Table 10), we find that while InQ is a strong determinant on its own, its moderating effect significantly amplifies the impact of EG on PDH, extending bidirectional causal links with both EG and PDH (Table 11). Specifically, reducing corruption, enhancing government efficiency, ensuring political stability, upholding the rule of law, and improving regulatory quality are instrumental in improving food security, productivity, and access to nutritious food. In practical terms, institutional quality operates through three key dimensions—transparency (regulatory quality and government effectiveness), accountability (voice and accountability and control of corruption), and participation (political stability and the rule of law). Each of these dimensions contributes synergically to enhancing a nation’s economic capacity and, consequently, its dietary habits. For SSA nations, adopting global best practices in good governance, as exemplified by developed nations, could significantly enhance institutional frameworks. Implementing robust anti-corruption measures, fostering transparency in public administration, and encouraging civic participation will create a more stable and effective governance environment [133]. This, in turn, will facilitate better economic policies, improve food distribution systems, and promote healthier dietary patterns across the region. These results are in line with prior studies by Azimi et al. [134], Nugroho et al. [135], Fotio et al. [136], and Jahangir et al. [137]. However, our findings demonstrate that UR negatively affects PDH, and InQ proves to substantially mitigate the negative effects of UR on the subject. Theoretically, these results suggest that existing economic instability, which primarily suppresses employment in both the public and private sectors, is foundational to reducing the financial capacity of the population to access necessary nutritious food. Weak InQ is another barrier to job creation. Therefore, macroeconomic policies aimed at mitigating unemployment and improving InQ would enhance household food security and promote healthier dietary patterns. Prior research by Milicic and DeCicca [37], Smed et al. [38], Bonaccio et al. [34], Colman and Dave [138], Hughes and Kumari [139], and Norström et al. [140] has also identified the negative impact of UR on PDH.

Furthermore, we examined the social impacts via SR. Our results (Table 7, Table 8, Table 9 and Table 10) indicate that while SR robustly improves PDH, the impact of elevated SR on PDH can be amplified through the modulation of InQ in the region. The causality analysis reported in Table 11 also substantiates a strong bidirectional causality relationship between SR and PDH. This association can be attributed to enhanced knowledge and nutrition literacy, which empower individuals to make healthier nutrition choices. Improved InQ can further bolster this impact by offering the required infrastructure and support systems to sustain these positive outcomes. These findings are consistent with those of Bashour [141], Huang et al. [142], Hadipour et al. [143], Guan et al. [144], and Aziz et al. [145], who have observed similar results. Likewise, GX demonstrates a positive association with PDH in SSA, indicating that increased government spending boosts sectoral performance and consequently improves dietary habits. Investments in livestock, education, and agricultural infrastructures foster access to nutritious foods, bolster agricultural productivity, and facilitate sufficient food availability initiatives. The significant moderating impact of InQ on the GX-PDH nexus underscores that higher institutional quality can enhance the effects of GX on PDH. This can be achieved through corruption control, improved government effectiveness, and reinforcement of the rule of law. These results align with those of prior studies, such as Kh’ng et al. [146], Faruk et al. [147], Tapsoba et al. [148], Onofrei et al. [149], Kim and Lane [150], Andersen et al. [151], Fani et al. [152], and Sunyal and Banerjee [153] that have consistently supported the beneficial effects of GX on PDH across diverse economies.

As an innovative approach, we captured the impact of GS, derived using the GARCH model from inflation rate data points, on PDH (Table 7). The results show that GS adversely affects PDH, while the moderating impact of the InQ significantly mitigates these negative consequences (Table 8, Table 9 and Table 10). The causality analysis reveals a unidirectional link from GS to PDH (Table 8). The theoretical framework supporting these results posits that inflationary shocks, perhaps, introduce uncertainties in the pricing of essential goods, disproportionately affecting vulnerable individuals who already allocate a tangible portion of their personal income to food. As GS increases, the real purchasing power diminishes, exacerbating food insecurity and poor dietary habits. However, robust institutional setups, such as stringent anti-corruption measures, effective governance, and the reinforcement of legal frameworks, can cushion this negative impact. These results are in line with prior studies, particularly those conducted by Gopakumar and Pandit [154], Olaoye et al. [155], Abaidoo and Agyapong [156], Munyenyembe et al. [157], Amporfu et al. [158], Sreenu [159], Tibrewal and Chaudhuri [160], and İlgün et al. [161]. Furthermore, our analysis reveals that FI has a detrimental effect on PDH, underscored by their bidirectional causal relationships. This implies that not only does FI impair PDH by restricting access to nutritious foods, but poor PDH can also exacerbate FI by leading to negative health outcomes that decrease people’s economic productivity [162]. However, the moderating impact of InQ is found to be effective in mitigating the negative effects of FI on PDH. Improved InQ enhances food distribution systems, performs social safety nets, and uplifts policies that facilitate access to essential foods. Our results align with the findings of Azimi and Rahman [105], Banerjee et al. [163], Ogunniyi et al. [164], Soko et al. [165], Kohanmoo et al. [41], Pereira and Hodge [166], Brown et al. [167], and Pasha et al. [168], who collectively demonstrated the negative impact of FI on PDH predictors.

Moreover, the findings support the positive association of URB with PDH, indicating that higher URB leads to better dietary habits. Urban environments often offer a wider range of food options, such as fresh produce and specialty healthy food items, which contribute to healthier dietary patterns. This association becomes even more robust with the modulation of InQ (Table 8, Table 9 and Table 10). Further assessment indicates that while URB causes PDH, improved PDH also promotes better URB. This bidirectional link suggests that as urban areas develop and offer more diverse food options, dietary habits improve, which in turn fosters further urban development and improvement of urban infrastructure and services. These results are consistent with prior research, including studies by Casari et al. [92], Ren et al. [52], Codjoe et al. [169], Popkin [13], Li and Lopez [170], Hovhannisyan and Devadoss [171], and Miao and Wu [172], which collectively supported the positive impact of URB on PDH. Finally, the findings demonstrate that GI positively impacts PDH, with the moderating impact of InQ improving their relationships. This implies that higher globalization facilitates greater access to essential food, diversifies food options, and promotes improvements in overall food quality. However, while the existing InQ is effective in enhancing the GI-PDH nexus, a strong commitment to uplifting InQ across SSA is essential to safely expand globalization through further trade liberalization, financial integration, international cooperation, and capital mobilization. The feedback response of PDH to FI further highlights that better PDH is essential to improve globalization across the SSA region. This is because improved PDH contributes to a more productive workforce, which in turn attracts higher foreign investments and improves further integration into the global economy. Recent studies by Kennedy et al. [173], Brunelle et al. [174], Hawkes [175], Bogin et al. [176], and Mendez and Popkin [177] have also found the positive influence of globalization on dietary habits across diverse economies.

## 7. Conclusions and Policy Implications

This study offers a comprehensive investigation of the multidimensional impact of economic growth, social factors, and demographic predictors on population dietary habits in a panel of 44 Sub-Saharan African nations. Employing a dataset from 2002 to 2022 and the PCSE and FGLS methods, the study aims to address the hitherto gaps in the literature, particularly the effects of inflationary shocks and the role of institutional quality on the diverse predictors of population dietary habits, including per capita protein intake, per capita fat intake, and per capita kilocalorie intake. While the initial results highlight significant nutritional deficiencies in SSA, they are compounded by recent inflationary shocks and existing poor institutional quality. The key findings indicate that economic growth, school enrollment rate, government expenditures, globalization, and urbanization positively influence population dietary habits. However, the unemployment rate, food insecurity, and inflationary shocks negatively impact the subject in SSA. As a counter predictor, the study employed the entropy method to construct an institutional quality index from the Worldwide Governance Indicators to gauge its impact both directly and as a mediating predictor. The results demonstrate that while institutional quality is positively associated with population dietary habits, its moderating role robustly promotes relationships between population dietary habits, economic growth, school enrollment rate, government expenditures, globalization, and urbanization. It also helps absorb the negative impact of inflationary shocks, the unemployment rate, and food insecurity on population dietary habits. The multidimensional causality nexus among variables further highlights key policy implications that are discussed as follows:

- Promoting institutional quality
- The study highlights the significant role of weak institutions in exacerbating social and economic issues, including poor dietary habits in SSA. Policymakers must initiate comprehensive reforms to combat elevated corruption, enhance governance efficiency, and improve political stability, all of which are shown to have a moderating effect on improving dietary habits. Specific measures include implementing inclusive digital governance platforms, introducing performance appraisal for public servants, and allocating resources for anti-corruption strategies. This can be achieved through advancing democratic administration, promoting inclusive political stability, and boosting training and education funding to build skilled human resources to facilitate effective reforms. These measures collectively boost sustainable growth, reduce corruption, and increase good governance, consequently overcoming dietary habit deficiencies in the region.
- Advancing economic resilience
- The study’s findings indicate that inflationary shocks and high unemployment rates negatively impact dietary habits across SSA. To address these issues, policymakers need to focus on creating macroeconomic stability by implementing strategies that mitigate the effects of inflation on food prices and unemployment on food access. Targeted subsidies for essential food items, the establishment of strategic food reserves, and the expansion of social safety nets are critical to protecting vulnerable populations from food insecurity during economic shocks. Additionally, policies aimed at promoting sustainable farming methods and economic diversification will help reduce the region’s reliance on volatile international markets, create jobs, and ensure a stable food supply chain. A well-rounded economic resilience strategy, coupled with strong institutional frameworks, will reduce the negative effects of inflation and unemployment on dietary habits.
- Enhancing dietary literacy
- The study reveals that higher school enrollment rates positively influence dietary habits, with institutional quality playing a crucial role in amplifying this effect. To capitalize on this, policymakers must prioritize improving access to quality education, particularly in rural areas. This involves investing in teacher training, modernizing school infrastructure, and ensuring that children, especially from vulnerable communities, are educated on proper nutrition. Incorporating comprehensive nutrition literacy into school curriculums will empower younger generations to adopt healthier dietary habits. Additionally, expanding school meal programs—offering free or subsidized meals—can directly address malnutrition and help instill long-term healthy dietary habits. These interventions will not only improve individual health but also contribute to broader improvements in population dietary habits across SSA. In addition to school-based interventions, public health campaigns and community-based initiatives that promote awareness of balanced diets and healthy eating practices are vital. Drawing on global best practices, these efforts should emphasize the importance of nutrition literacy and create an environment conducive to healthier living across SSA.
- Globalization enhancement
- Although the existing globalization index score is below 50 in the SSA, its impact on population dietary habits remains significantly positive. This suggests that further enhancement of globalization could lead to improved dietary habits in the region by facilitating greater access to essential foods and diversifying food options. Policymakers need to prioritize economic, political, and social strategies to achieve this. For instance, reducing tariffs and minimizing trade barriers can increase the flow of goods and services across borders. Additionally, human capital exchange agreements, which promote educational programs, cultural exchange, and global cooperation, can collectively enhance dietary habits across the region.

### Limitations

Like all empirical studies, the present investigation has two key limitations, though the overall reliability and comprehensiveness of its results remain valid. Firstly, the unavailability of sufficient and consecutive time series datasets for the required variables led to the exclusion of several countries from the analysis. This constraint may have limited the overall generalizability of the findings across the entire SSA region, as the excluded countries might exhibit different economic, social, and institutional dynamics that could influence the relationship between the studied variables and dietary habits. Secondly, due to issues of over-specification and multicollinearity, we had to exclude two key variables—foreign direct investment (FDI) and population growth rate—from the final analysis. These variables, while theoretically relevant, could not be reliably integrated into the model without distorting the results. Excluding FDI may overlook its potential role in shaping economic resilience and access to food supplies, particularly in SSA, while population growth could significantly impact food demand, unemployment rates, and social services, all of which influence dietary habits. Future studies could address these empirical challenges by using larger datasets or alternative modeling approaches to handle multicollinearity or expanding the scope of the analysis to include different economic conditions. This would allow for a more robust understanding of the interactions between FDI, population growth, and dietary habits. Additionally, while the study employed advanced econometric methods to ensure the robustness of its findings, there are inherent limitations in relying on secondary data, which may contain biases or measurement errors that could affect the reliability of the conclusions. As a result, future research could complement quantitative analysis with qualitative case studies or mixed-method approaches to capture context-specific dynamics and provide deeper insights into the socio-economic and institutional factors influencing dietary habits in the region.

## Figures and Tables

**Table 2:** Descriptive statistics.

Variable	Obs.	Mean	Std. Dev.	Minimum	Maximum
PP	924	41.443	14.049	23.410	67.940
PF	924	33.636	16.909	13.700	47.240
PK	924	2079.19	328.797	1522.11	2387.000
EG	924	1881.997	2264.555	255.100	14,222.550
UR	924	8.180	6.908	0.320	37.850
SR	924	99.722	11.374	45.400	144.730
GX	924	3.757	1.110	0.620	7.400
GS	924	1.623	27.368	−28.840	597.280
InQ	924	0.343	0.217	0.000	0.892
FI	924	21.994	13.410	2.700	70.900
URB	924	39.971	16.431	8.680	90.740
GI	924	46.649	3.076	40.230	49.728

List of countries: Angola, Benin, Botswana, Burkina Faso, Burundi, Cabo Verde, Cameroon, Central African Republic, Chad, Comoros, Congo DEM, Congo REP, Côte d‘Ivoire, Equatorial Guinea, Eswatini, Ethiopia, Gabon, Gambia, Ghana, Guinea, Guinea-Bissau, Kenya, Lesotho, Liberia, Madagascar, Malawi, Mali, Mauritania, Mauritius, Mozambique, Namibia, Niger, Nigeria, Rwanda, Senegal, Sierra Leone, Somalia, South Africa, Sudan, Tanzania, Togo, Uganda, Zambia, Zimbabwe.

**Table 3:** Correlation matrix.

Variables	Correlation Analysis												VIF	
	PP	PF	PK	EG	UR	SR	GX	GS	InQ	FI	URB	GI	Stat.	1/VIF
PP	1.00													
PF	0.62	1.00												
PK	0.76	0.65	1.00											
EG	0.26	0.25	0.15	1.00									3.57	0.28
UR	0.27	0.23	0.17	0.44	1.00								1.64	0.61
SR	−0.04	−0.03	0.11	0.03	−0.06	1.00							1.05	0.95
GX	0.24	0.14	0.27	0.36	0.46	−0.07	1.00						1.35	0.74
GS	−0.06	0.07	−0.07	−0.04	0.03	−0.04	0.02	1.00					1.02	0.97
InQ	0.22	0.09	0.18	0.40	0.23	0.03	0.14	−0.11	1.00				1.26	0.79
FI	−0.29	−0.12	−0.38	−0.36	0.04	−0.19	−0.03	0.07	−0.27	1.00			1.35	0.73
URB	0.11	0.37	0.25	0.56	0.37	−0.02	0.27	−0.04	0.16	−0.15	1.00		1.57	0.63
GI	0.24	0.27	0.18	0.82	0.49	0.05	0.38	−0.02	0.34	−0.34	0.54	1.00	3.43	0.29

Notes: VIF: variance inflation factor. Mean VIF = 1.80.

**Table 4:** Cross-sectional dependence and slope heterogeneity results.

Cross-Sectional Dependence Test	CD-Test	*p*-Value	Corr	Abs (corr)
PP	46.23 ***	0.000	0.328	0.558
PF	29.19 ***	0.000	0.207	0.439
PK	35.67 ***	0.000	0.253	0.587
EG	56.46 ***	0.000	0.401	0.624
UR	16.30 ***	0.000	0.116	0.473
SR	8.88 ***	0.000	0.063	0.462
GX	14.39 ***	0.000	0.102	0.344
GS	17.32 ***	0.000	0.123	0.235
InQ	1.05	0.293	0.007	0.421
FI	20.22 ***	0.000	0.143	0.541
URB	112.20 ***	0.000	0.796	0.962
GI	32.23 ***	0.000	0.229	0.523
Slope heterogeneity test	Delta	*p*-value	Adj-delta	*p*-value
Model 1–PP	11.593 ***	0.000	16.800 ***	0.000
Model 2–PF	9.686 ***	0.000	14.036 ***	0.000
Model 3–PK	9.154 ***	0.000	13.265 ***	0.000

Notes: *** rejects the null hypothesis at the 1% significance level.

**Table 5:** Panel unit root test results.

Variables	CADF Test		CIPS Test	
	Level	First-diff.	Level	First-diff.
	t-stat.	t-stat.	Test-stat.	Test-stat.
PP	−2.196	−3.317 ***	−2.101	−4.026 ***
PF	−1.921	−3.404 ***	−2.519	−4.704 ***
PK	−2.084	−2.991 ***	−2.683	−4.689 ***
EG	−2.116	−2.665 ***	−2.168	−3.603 ***
UR	−2.156	−2.769 ***	−1.901	−3.042 ***
SR	−2.467	−2.765 ***	−2.010	−3.182 ***
GX	−3.598 ***	−3.859 ***	−3.086 ***	−5.019 ***
GS	−3.111 ***	−3.877 ***	−3.965 ***	−5.432 ***
InQ	−2.907 ***	−3.113 ***	−2.796 ***	−4.474 ***
FI	−2.140	−2.869 ***	−1.254	−3.421 ***
URB	−1.567	−3.888 ***	−1.597	−3.510 ***
GI	−2.154	−2.908 ***	−2.255	−4.332 ***

Notes: *** rejects null hypothesis at the 1% significance level.

**Table 6:** Panel cointegration results.

Models Estimated	Model 1–PP		Model 2–PF		Model 3–PK	
	Statistics	*p*-Value	Statistics	*p*-Value	Statistics	*p*-Value
***Pedroni’s results***						
Modified variance ratio	−5.805 ***	0.000	−5.361 ***	0.000	−5.247 ***	0.000
Modified Phillips–Perron t	4.676 ***	0.000	4.762 ***	0.000	5.639 ***	0.000
Phillips–Perron t	−7.679 ***	0.000	−5.191 ***	0.000	−3.619 ***	0.001
Augmented Dickey–Fuller t	−6.425 ***	0.000	−4.186 ***	0.000	−3.923 ***	0.000
***Westerlund’s results***						
Variance ratio	−1.584 *	0.056	−1.803 **	0.038	−1.665 **	0.047

Notes: ***, **, and * reject null hypothesis at the 1%, 5%, and 10% significance levels, respectively.

**Table 7:** Direct unconditional results.

Variables	PCSE Model Estimates			FGLS Model Estimates		
	Model 1–PP	Model 1–PF	Model 1–PK	Model 1–PP	Model 1–PF	Model 1–PK
EG	0.1884 *** (4.289)	0.2985 *** (4.416)	0.443 *** (5.283)	0.1884 *** (4.011)	0.2985 *** (4.117)	0.443 *** (5.617)
UR	−0.449 *** (−8.600)	−0.274 *** (−7.742)	−0.137 *** (−6.818)	−0.449 *** (−5.880)	−0.274 *** (−2.897)	−0.137 *** (−3.051)
SR	0.122 *** (3.758)	0.053 * (1.952)	1.485 *** (4.629)	0.122 *** (3.294)	0.053 *** (3.156)	1.485 * (1.817)
GX	1.948 *** (8.944)	0.131 *** (3.419)	1.559 *** (12.882)	1.948 *** (4.513)	0.131 *** (3.246)	1.559 *** (8.465)
GS	−0.524 *** (−2.577)	−0.513 *** (−3.638)	−0.568 *** (−2.700)	−0.524 ** (−2.571)	0.513 *** (2.675)	−0.568 * (−1.692)
InQ	4.521 *** (4.514)	2.064 *** (4.056)	4.661 *** (6.037)	4.521 ** (2.117)	2.064 *** (4.024)	4.661 *** (2.839)
FI	−0.329 *** (−18.547)	−0.120 *** (−3.327)	−1.593 *** (−18.972)	−0.329 *** (−9.206)	−0.120 *** (−2.720)	−1.593 *** (−13.432)
URB	0.059 *** (2.715)	0.326 *** (12.352)	0.178 *** (11.489)	0.059 * (1.890)	0.326 *** (8.381)	5.178 *** (7.471)
GI	0.606 ** (2.232)	0.720 ** (2.477)	0.678 *** (3.235)	0.606 *** (3.142)	0.720 *** (3.097)	0.678 *** (4.998)
Constant	70.570 *** (20.079)	46.125 *** (16.319)	57.038 *** (49.111)	70.570 *** (16.172)	46.125 *** (8.542)	57.038 *** (20.341)
***Post-estimations***						
Observations	924	924	924	924	924	924
R-squared	0.617	0.555	0.498	0.621	0.644	0.512
Number of units	44	44	44	44	44	44

Notes: z-statistics are presented in parenthesis. ***, **, and * indicate significance at 1%, 5%, and 10% levels, respectively.

**Table 8:** Moderating effects of InQ on PP-Xs nexus.

Variables	PCSE Model Estimates							
	InQ on PP–EG Nexus	InQ on PP–UR Nexus	InQ on PP–SR Nexus	InQ on PP–GX Nexus	InQ on PP–GS Nexus	InQ on PP–FI Nexus	InQ on PP–URB Nexus	InQ on PP–FI Nexus
EG	0.1878 ***	0.1709 ***	0.1825 ***	0.1893 ***	0.1799 ***	0.1862 **	0.1891 **	0.1835 ***
	(6.33)	(3.40)	(2.88)	(3.34)	(3.57)	(2.44)	(2.52)	(9.25)
UR	−0.406 ***	−0.442 ***	−0.445 ***	−0.477 ***	−0.448 ***	−0.467 ***	−0.483 ***	−0.364 ***
	(−9.36)	(−7.38)	(−8.59)	(−9.29)	(−7.95)	(−6.61)	(−9.12)	(−7.20)
SR	0.142 ***	0.145 ***	0.160 ***	0.141 ***	0.134 ***	0.142 ***	0.133 ***	0.146 ***
	(4.25)	(3.45)	(4.92)	(3.90)	(3.91)	(3.55)	(3.89)	(4.27)
GX	0.176 ***	0.195 ***	0.194 ***	0.103 ***	0.154 ***	0.191 ***	0.171 ***	0.199 ***
	(10.58)	(8.27)	(8.57)	(7.76)	(8.53)	(10.44)	(8.59)	(10.85)
GS	−0.528 ***	−0.526 ***	−0.524 ***	−0.523 **	−0.536 ***	−0.522 **	−0.528 ***	−0.522 **
	(−2.91)	(−2.72)	(−2.62)	(−2.49)	(−5.29)	(−2.37)	(−2.93)	(−2.47)
InQ	4.927 ***	4.383 ***	4.436 ***	4.105 ***	4.444 ***	4.228 ***	4.200 ***	4.510 ***
	(3.12)	(3.37)	(3.97)	(3.25)	(3.16)	(6.83)	(8.64)	(4.70)
FI	−0.430 ***	−0.316 ***	−0.354 ***	−0.336 ***	−0.331 ***	−0.350 **	−0.367 ***	−0.394 ***
	(−8.55)	(−6.87)	(−8.67)	(−7.49)	(−8.27)	(−2.29)	(−3.36)	(−8.49)
URB	0.044 ***	0.082 ***	0.058 ***	0.054 **	0.060 ***	0.024 ***	0.055 ***	0.046 ***
	(2.65)	(3.68)	(2.79)	(2.36)	(2.79)	(3.31)	(7.96)	(2.79)
GI	0.821 ***	0.480 ***	0.637 **	0.332 ***	0.550 **	0.484 ***	0.393 ***	0.431 ***
	(3.96)	(3.69)	(2.39)	(2.77)	(2.07)	(10.24)	(5.96)	(10.79)
InQ × EG	1.047 ***							
	(4.37)							
InQ × UR		−0.087 ***						
		(−9.57)						
InQ × SR			1.752 ***					
			(4.31)					
InQ × GX				2.396 ***				
				(8.57)				
InQ × GS					−0.00058 ***			
					(−5.79)			
InQ × FI						−0.0007 ***		
						(−15.38)		
InQ × URB							0.832 ***	
							(9.354)	
InQ × GI								1.00014 ***
								(7.95)
Constant	79.378 ***	65.417 ***	94.005 ***	60.146 ***	71.392 ***	58.194 ***	84.998 ***	76.554 ***
	(22.59)	(19.23)	(12.98)	(17.63)	(20.21)	(20.17)	(18.11)	(20.64)
***Post-estimations***								
Observations	924	924	924	924	924	924	924	924
R-squared	0.326	0.230	0.216	0.235	0.232	0.327	0.236	0.305
Number of ID	44	44	44	44	44	44	44	44

Notes: z-statistics are presented in parenthesis. ***, **, and indicate significance at 1%, 5% levels, respectively.

**Table 9:** Moderating effects of InQ on PF-Xs nexus.

Variables	PCSE Model Estimates							
	InQ on PF–EG Nexus	InQ on PF–UR Nexus	InQ on PF–SR Nexus	InQ on PF–GX Nexus	InQ on PF–GS Nexus	InQ on PF–FI Nexus	InQ on PF–URB Nexus	InQ on PF–GI Nexus
EG	0.2927 ***	0.2841 **	0.2799 **	0.2833 ***	0.2785 **	0.2789 ***	0.2872 ***	0.2915 ***
	(20.60)	(2.03)	(2.27)	(2.92)	(2.25)	(4.81)	(3.14)	(9.37)
UR	−0.267 ***	−0.259 ***	−0.262 ***	−0.301 ***	−0.272 ***	−0.300 ***	−0.319 ***	−0.286 ***
	(−4.89)	(−8.43)	(−7.39)	(−8.38)	(−6.88)	(−7.62)	(−9.08)	(−2.75)
SR	0.058 ***	0.049 *	0.055 ***	0.072 ***	0.071 **	0.023	0.067 **	0.098 ***
	(3.06)	(1.86)	(6.13)	(2.58)	(2.47)	(0.820)	(2.04)	(3.64)
GX	1.003 ***	1.033 **	1.808 **	1.122 ***	1.431 ***	1.195 ***	1.293 **	1.706 ***
	(5.32)	(2.10)	(2.48)	(5.37)	(1.34)	(0.65)	(2.27)	(5.21)
GS	−0.505 ***	−0.511 ***	−0.512 ***	−0.514 ***	−0.544 ***	−0.516 ***	−0.507 ***	−0.516 ***
	(−3.31)	(−3.55)	(−3.59)	(−2.68)	(−5.03)	(−2.73)	(−3.39)	(−2.80)
InQ	2.185 ***	2.553 ***	2.905 ***	2.666 ***	2.627 ***	2.986 ***	2.899 ***	2.499 ***
	(4.04)	(4.78)	(5.87)	(5.83)	(3.45)	(6.56)	(4.01)	(6.81)
FI	−0.222 ***	−0.212 ***	−0.287 ***	−0.227 ***	−0.223 ***	−0.239 ***	−0.270 ***	−0.240 ***
	(−4.02)	(−2.89)	(−5.31)	(−3.77)	(−3.24)	(−7.03)	(−4.91)	(−8.38)
URB	0.306 ***	0.311 ***	0.330 ***	0.331 ***	0.326 ***	0.379 ***	0.363 *	0.407 ***
	(5.02)	(6.634)	(4.677)	(6.638)	(4.224)	(5.364)	(6.692)	(8.648)
GI	0.735 ***	0.727 ***	0.635 **	0.745 ***	0.802 ***	0.704 ***	0.713	0.824 ***
	(3.21)	(3.75)	(2.11)	(3.65)	(2.72)	(5.46)	(3.19)	(7.64)
InQ × EG	1.215 ***							
	(6.89)							
InQ × UR		−0.097 ***						
		(−4.89)						
InQ × SR			2.064 ***					
			(5.80)					
InQ × GX				1.187 ***				
				(5.92)				
InQ × GS					−0.0009 ***			
					(−5.45)			
InQ × FI						−0.084 ***		
						(−5.38)		
InQ × URB							1.093 ***	
							(11.27)	
InQ × GI								1.006 ***
								(3.14)
Constant	61.889 ***	42.774 ***	110.482 ***	35.911 ***	47.318 ***	27.903 ***	65.068 ***	57.205 ***
	(19.46)	(15.96)	(9.92)	(10.82)	(16.27)	(8.13)	(16.46)	(19.90)
Observations	924	924	924	924	924	924	924	924
R-squared	0.428	0.167	0.215	0.179	0.199	0.342	0.197	0.397
Number of ID	44	44	44	44	44	44	44	44

Notes: z-statistics are presented in parenthesis. ***, **, and * indicate significance at 1%, 5%, and 10% levels, respectively.

**Table 11:** Granger non-causality test results.

Direction of Causality	Model 1–PP			Model 2–PF			Model 3–PK		
	W-bar	Z-bar	*p*-Value	W-bar	Z-bar	*p*-Value	W-bar	Z-bar	*p*-Value
EG → PDH	4.511	16.471 ***	0.000	2.330	6.239 ***	0.000	3.730	12.807 ***	0.000
UR → PDH	2.377	6.461 ***	0.000	2.166	5.470 ***	0.000	2.116	5.234 ***	0.000
SR → PDH	2.786	8.379 ***	0.000	2.783	8.365 ***	0.000	3.782	13.048 ***	0.000
GX → PDH	2.876	8.799 ***	0.000	2.465	6.875 ***	0.000	2.432	6.720 ***	0.000
GS → PDH	1.501	2.353 **	0.018	2.874	5.586 ***	0.000	2.324	6.585 ***	0.000
InQ → PDH	3.628	12.327 ***	0.000	2.267	5.945 ***	0.000	3.068	9.699 ***	0.000
FI → PDH	7.667	31.275	0.000	6.990	28.097 ***	0.000	17.819	78.888 ***	0.000
URB → PDH	6.289	24.811 ***	0.000	3.942	13.802 ***	0.000	6.028	23.587 ***	0.000
FI → PDH	2.647	7.727 ***	0.000	2.547	7.258 ***	0.000	2.985	9.311 ***	0.000
***Feedback response***									
EG ← PDH	3.410	11.308 ***	0.000	2.851	8.684 ***	0.000	3.188	10.266 ***	0.000
UR ← PDH	2.242	5.829 ***	0.000	2.688	7.919 ***	0.000	1.814	3.822 ***	0.000
SR ← PDH	2.682	7.891 ***	0.000	2.224	5.744 ***	0.000	2.183	5.549 ***	0.000
GX ← PDH	2.934	9.073 ***	0.000	1.391	1.837 *	0.066	1.860	4.033 ***	0.000
GS ← PDH	1.227	1.067	0.285	0.987	−0.056	0.954	1.359	1.487	0.191
InQ ← PDH	1.493	2.313 **	0.020	1.925	4.340 ***	0.000	1.821	3.850 ***	0.000
FI ← PDH	6.030	23.593 ***	0.000	3.157	10.119 ***	0.000	13.853	60.287 ***	0.000
URB ← PDH	5.872	22.854 ***	0.000	3.121	9.948 ***	0.000	3.178	10.218 ***	0.000
FI ← PDH	2.305	6.120 ***	0.000	2.374	6.449 ***	0.000	2.135	5.325 ***	0.000

Notes: ***, **, and * indicate significance at 1%, 5%, and 10% levels, respectively.

## Data Availability

The datasets used in the study are publicly available at the World Bank, UN-FAO, and KOF Swiss Economic Institute’s websites.

## Appendix A

**STATA codes for GS construction**

*****Generate Inflationary Shock variable *****

arch inf, arch (1) garch(1)///inf = inflation rate

predict shock, variance

predict GS, resid

## Appendix B

**STATA codes for InQ construction**

******Normalize VC *****

egen min_VC = min(VC)

egen max_VC = max(VC)

gen Z_VC = (VC − min_VC)/(max_VC − min_VC)

*****Normalize PS *****

egen min_PS = min(PS)

egen max_PS = max(PS)

gen Z_PS = (PS − min_PS)/(max_PS − min_PS)

*****Normalize GE *****

egen min_GE = min(GE)

egen max_GE = max(GE)

gen Z_GE = (GE − min_GE)/(max_GE − min_GE)

*****Normalize RQ *****

egen min_RQ = min(RQ)

egen max_RQ = max(RQ)

gen Z_RQ = (RQ − min_RQ)/(max_RQ − min_RQ)

*****Normalize CC *****

egen min_CC = min(CC)

egen max_CC = max(CC)

gen Z_CC = (CC − min_CC)/(max_CC − min_CC)

*****Normalize RL *****

egen min_RL = min(RL)

egen max_RL = max(RL)

gen Z_RL = (RL − min_RL)/(max_RL − min_RL)

*****Calculate proportions for normalized indicators *****

egen sum_Z_VC = total(Z_VC)

egen sum_Z_PS = total(Z_PS)

egen sum_Z_GE = total(Z_GE)

egen sum_Z_RQ = total(Z_RQ)

egen sum_Z_CC = total(Z_CC)

egen sum_Z_RL = total(Z_RL)

gen p_VC = Z_VC/sum_Z_VC

gen p_PS = Z_PS/sum_Z_PS

gen p_GE = Z_GE/sum_Z_GE

gen p_RQ = Z_RQ/sum_Z_RQ

gen p_CC = Z_CC/sum_Z_CC

gen p_RL = Z_RL/sum_Z_RL

*****Calculate entropy for each indicator *****

gen e_VC = −p_VC * log(p_VC)

gen e_PS = −p_PS * log(p_PS)

gen e_GE = −p_GE * log(p_GE)

gen e_RQ = −p_RQ * log(p_RQ)

gen e_CC = −p_CC * log(p_CC)

gen e_RL = −p_RL * log(p_RL)

*****Sum entropy across all observations for each indicator *****

egen sum_e_VC = total(e_VC)

egen sum_e_PS = total(e_PS)

egen sum_e_GE = total(e_GE)

egen sum_e_RQ = total(e_RQ)

egen sum_e_CC = total(e_CC)

egen sum_e_RL = total(e_RL)

*****Calculate weights for each indicator *****

gen w_VC = (1 − sum_e_VC)/(6 − (sum_e_VC + sum_e_PS + sum_e_GE + sum_e_RQ + sum_e_CC + sum_e_RL))

gen w_PS = (1 − sum_e_PS)/(6 − (sum_e_VC + sum_e_PS + sum_e_GE + sum_e_RQ + sum_e_CC + sum_e_RL))

gen w_GE = (1 − sum_e_GE)/(6 − (sum_e_VC + sum_e_PS + sum_e_GE + sum_e_RQ + sum_e_CC + sum_e_RL))

gen w_RQ = (1 − sum_e_RQ)/(6 − (sum_e_VC + sum_e_PS + sum_e_GE + sum_e_RQ + sum_e_CC + sum_e_RL))

gen w_CC = (1 − sum_e_CC)/(6 − (sum_e_VC + sum_e_PS + sum_e_GE + sum_e_RQ + sum_e_CC + sum_e_RL))

gen w_RL = (1 − sum_e_RL)/(6 − (sum_e_VC + sum_e_PS + sum_e_GE + sum_e_RQ + sum_e_CC + sum_e_RL))

*****Construct the Institutional Quality Index (InQ) *****

gen InQ = w_VC * Z_VC + w_PS * Z_PS + w_GE * Z_GE + w_RQ * Z_RQ + w_CC * Z_CC + w_RL * Z_RL

## Appendix C

FGLS model estimates: robustness tests for Table 8, Table 9 and Table 10.

**Table A1:** Moderating effects of InQ on PP-Xs nexus.

Variables	FGLS Model Estimates							
	InQ on PP–EG Nexus	InQ on PP–UR Nexus	InQ on PP–SR Nexus	InQ on PP–GX Nexus	InQ on PP–GS Nexus	InQ on PP–FI Nexus	InQ on PP–URB Nexus	InQ on PP–FI Nexus
EG	0.1878 ***	0.1709 ***	0.1825 ***	0.1893 ***	0.1799 ***	0.1862 **	0.1891 **	0.1835 ***
	(9.12)	(5.02)	(4.16)	(3.88)	(5.10)	(2.13)	(2.49)	(8.37)
UR	−0.406 ***	−0.442 ***	−0.445 ***	−0.477 ***	−0.448 ***	−0.467 ***	−0.483 ***	−0.364 ***
	(−9.36)	(−7.38)	(−8.59)	(−9.29)	(−7.95)	(−6.61)	(−9.12)	(−7.20)
SR	0.142 ***	0.145 ***	0.160 ***	0.141 ***	0.134 ***	0.142 ***	0.133 ***	0.146 ***
	(4.25)	(3.45)	(4.92)	(3.90)	(3.91)	(3.55)	(3.89)	(4.27)
GX	0.176 ***	0.195 ***	0.194 ***	0.103 ***	0.154 ***	0.191 ***	0.171 ***	0.199 ***
	(10.58)	(8.27)	(8.57)	(7.76)	(8.53)	(10.44)	(8.59)	(10.85)
GS	−0.528 ***	−0.526 ***	−0.524 ***	−0.523 **	−0.536 ***	−0.522 **	−0.528 ***	−0.522 **
	(−4.86)	(−5.39)	(−4.81)	(−2.33)	(−5.70)	(−2.11)	(−4.32)	(−2.45)
InQ	4.927 ***	4.383 ***	4.436 ***	4.105 ***	4.444 ***	4.228 ***	4.200 ***	4.510 ***
	(3.12)	(3.37)	(3.97)	(3.25)	(3.16)	(6.83)	(8.64)	(4.70)
FI	−0.430 ***	−0.316 ***	−0.354 ***	−0.336 ***	−0.331 ***	−0.350 **	−0.367 ***	−0.394 ***
	(−8.55)	(−6.87)	(−8.67)	(−7.49)	(−8.27)	(−2.29)	(−3.36)	(−8.49)
URB	0.044 ***	0.082 ***	0.058 ***	0.054 **	0.060 ***	0.024 ***	0.055 ***	0.046 ***
	(2.65)	(3.68)	(2.79)	(2.36)	(2.79)	(3.31)	(7.96)	(2.79)
GI	0.821 ***	0.480 ***	0.637 **	0.332 ***	0.550 **	0.484 ***	0.393 ***	0.431 ***
	(3.96)	(3.69)	(2.39)	(2.77)	(2.07)	(10.24)	(5.96)	(10.79)
InQ × EG	1.047 ***							
	(5.66)							
InQ × UR		−0.087 ***						
		(−11.49)						
InQ × SR			1.752 ***					
			(7.15)					
InQ × GX				2.396 ***				
				(8.11)				
InQ × GS					−0.00058 ***			
					(−7.27)			
InQ × FI						−0.0007 ***		
						(−15.41)		
InQ × URB							0.832 ***	
							(10.28)	
InQ × GI								1.00014 ***
								(9.12)
Constant	79.378 ***	65.417 ***	94.005 ***	60.146 ***	71.392 ***	58.194 ***	84.998 ***	76.554 ***
	(22.59)	(19.23)	(12.98)	(17.63)	(20.21)	(20.17)	(18.11)	(20.64)
***Post-estimations***								
Observations	924	924	924	924	924	924	924	924
Number of ID	0.326	0.230	0.216	0.235	0.232	0.327	0.236	0.305

Notes: z-statistics are presented in parenthesis. ***, **, and indicate significance at 1%, 5% levels, respectively.

**Table A2:** Moderating effects of InQ on PF-Xs nexus.

Variables	FGLS Model Estimates							
	InQ on PF–EG Nexus	InQ on PF–UR Nexus	InQ on PF–SR Nexus	InQ on PF–GX Nexus	InQ on PF–GS Nexus	InQ on PF–FI Nexus	InQ on PF–URB Nexus	InQ on PF–GI Nexus
EG	0.2927 ***	0.2841 **	0.2799 **	0.2833 ***	0.2785 **	0.2789 ***	0.2872 ***	0.2915 ***
	(20.60)	(2.03)	(2.27)	(2.92)	(2.25)	(4.81)	(3.14)	(9.37)
UR	−0.267 ***	−0.259 ***	−0.262 ***	−0.301 ***	−0.272 ***	−0.300 ***	−0.319 ***	−0.286 ***
	(−4.89)	(−8.43)	(−7.39)	(−8.38)	(−6.88)	(−7.62)	(−9.08)	(−2.75)
SR	0.058 ***	0.049 ***	0.055 ***	0.072 ***	0.071 ***	0.023 ***	0.067 ***	0.098 ***
	(3.06)	(4.15)	(6.13)	(2.58)	(3.99)	(4.18)	(5.17)	(3.64)
GX	1.003 ***	1.033 **	1.808 **	1.122 ***	1.431 ***	1.195 ***	1.293 **	1.706 ***
	(5.32)	(2.10)	(2.48)	(5.37)	(1.34)	(0.65)	(2.27)	(5.21)
GS	−0.505 ***	−0.511 ***	−0.512 ***	−0.514 ***	−0.544 ***	−0.516 ***	−0.507 ***	−0.516 ***
	(−3.31)	(−3.55)	(−3.59)	(−2.68)	(−5.03)	(−2.73)	(−3.39)	(−2.80)
InQ	2.185 ***	2.553 ***	2.905 ***	2.666 ***	2.627 ***	2.986 ***	2.899 ***	2.499 ***
	(4.04)	(4.78)	(5.87)	(5.83)	(3.45)	(6.56)	(4.01)	(6.81)
FI	−0.222 ***	−0.212 ***	−0.287 ***	−0.227 ***	−0.223 ***	−0.239 ***	−0.270 ***	−0.240 ***
	(−4.02)	(−2.89)	(−5.31)	(−3.77)	(−3.24)	(−7.03)	(−4.91)	(−8.38)
URB	0.306 ***	0.311 ***	0.330 ***	0.331 ***	0.326 ***	0.379 ***	0.363 *	0.407 ***
	(5.02)	(6.634)	(4.677)	(6.638)	(4.224)	(5.364)	(6.692)	(8.648)
GI	0.735 ***	0.727 ***	0.635 **	0.745 ***	0.802 ***	0.704 ***	0.713	0.824 ***
	(3.21)	(3.75)	(2.11)	(3.65)	(2.72)	(5.46)	(3.19)	(7.64)
InQ × EG	1.215 ***							
	(6.89)							
InQ × UR		−0.097 ***						
		(-8.09)						
InQ × SR			2.064 ***					
			(6.13)					
InQ × GX				1.187 ***				
				(7.88)				
InQ × GS					−0.0009 ***			
					(−6.10)			
InQ × FI						−0.084 ***		
						(−6.17)		
InQ × URB							1.093 ***	
							(13.00)	
InQ × GI								1.006 ***
								(5.99)
Constant	61.889 ***	42.774 ***	110.482 ***	35.911 ***	47.318 ***	27.903 ***	65.068 ***	57.205 ***
	(20.02)	(13.44)	(10.38)	(11.67)	(18.11)	(7.98)	(18.12)	(20.17)
***Post-estimations***								
Observations	924	924	924	924	924	924	924	924
Number of ID	44	44	44	44	44	44	44	44

Notes: z-statistics are presented in parenthesis. ***, **, and * indicate significance at 1%, 5%, and 10% levels, respectively.

**Table A3:** Moderating effects of InQ on PK-Xs nexus.

Variables	FGLS Model Estimates							
	InQ on PK–EG Nexus	InQ on PK–UR Nexus	InQ on PK–SR Nexus	InQ on PK–GX Nexus	InQ on PK–GS Nexus	InQ on PK–FI Nexus	InQ on PK–URB Nexus	InQ on PK–CO_2_e Nexus
EG	0.454 ***	0.448 ***	0.445 ***	0.452 ***	0.445 ***	0.436 ***	0.438 ***	0.447 ***
	(20.49)	(6.43)	(5.56)	(7.05)	(5.50)	(5.74)	(4.76)	(4.46)
UR	−0.147 ***	−0.147 ***	−0.158 ***	−0.177 ***	−0.123 ***	−0.174 ***	−0.1637 ***	−0.146 ***
	(−5.93)	(−5.14)	(−6.62)	(−7.80)	(−6.38)	(−4.23)	(−7.44)	(−3.15)
SR	1.409 ***	1.649 ***	1.772 ***	1.750 ***	1.7311 ***	1.7164 ***	1.8326 ***	1.719 **
	(3.07)	(4.69)	(3.00)	(2.76)	(4.01)	(7.61)	(4.04)	(2.28)
GX	1.082 ***	1.451 ***	1.287 ***	1.311 ***	1.470 ***	1.010 ***	1.350 ***	1.343 ***
	(13.09)	(12.45)	(12.02)	(11.20)	(12.31)	(16.80)	(12.32)	(13.69)
GS	−0.596 **	−0.626 ***	−0.572 ***	−0.549 **	−0.684 ***	−0.498 ***	−0.633 ***	−0.510 ***
	(−2.47)	(−2.95)	(−2.78)	(−2.53)	(−3.25)	(−2.85)	(−2.71)	(−2.58)
InQ	4.089 ***	4.034 ***	4.198 ***	4.077 ***	4.061 ***	4.017 ***	4.110 ***	4.094 ***
	(8.36)	(10.79)	(4.31)	(7.54)	(5.51)	(9.92)	(7.29)	(4.40)
FI	−1.589 ***	−1.582 ***	−1.560 ***	−1.752 ***	−1.620 ***	−1.206 **	−1.546 ***	−1.628 ***
	(−5.81)	(−6.55)	(−5.25)	(−7.97)	(−9.62)	(−4.30)	(−8.59)	(−5.05)
URB	0.181 ***	0.182 ***	0.208 ***	0.206 ***	0.177 ***	0.185 ***	0.181 *	0.149 ***
	(6.48)	(9.78)	(6.18)	(6.11)	(9.70)	(9.29)	(1.67)	(7.15)
GI	0.667 **	0.594 *	0.677 **	0.786 **	0.887 ***	0.879 ***	0.804 **	0.856 ***
	(2.41)	(1.73)	(1.31)	(2.32)	(3.14)	(9.53)	(2.53)	(8.17)
InQ × EG	0.949 ***							
	(5.13)							
InQ × UR		−0.0025 ***						
		(−9.10)						
InQ × SR			2.422 ***					
			(4.80)					
InQ × GX				2.857 ***				
				(6.97)				
InQ × GS					−0.0332 ***			
					(−4.51)			
InQ × FI						−0.00871 ***		
						(−6.83)		
InQ × URB							1.080 ***	
							(8.37)	
InQ × GI								1.0605 ***
								(4.95)
Constant	8.147 ***	2.359 ***	6.654 ***	8.549 ***	8.636 ***	9.900 ***	6.482 ***	5.458 ***
	(79.43)	(42.21)	(24.31)	(28.80)	(51.10)	(33.18)	(46.77)	(65.80)
***Post-estimations***								
Observations	924	924	924	924	924	924	924	924
Number of ID	44	44	44	44	44	44	44	44

Notes: z-statistics are presented in parenthesis. ***, **, and * indicate significance at 1%, 5%, and 10% levels, respectively.
